# An all-in-one injectable biocement: self-setting magnesium phosphate for bone repair, fracture adhesion and osteoporotic fixation

**DOI:** 10.1093/rb/rbaf133

**Published:** 2025-12-27

**Authors:** Xinyu Qu, Mengting Yin, Zhongyi Sun, Zhang Liu, Jing Ru, Haibo Liu, Rui Xu, Olga Musskaya, Ilya Glazov, Bingqiang Lu, Xinyu Zhao, Bingdi Chen, Anatoly Kulak, Feng Chen

**Affiliations:** Center for Orthopaedic Science and Translational Medicine, Department of Orthopedics, Shanghai Tenth People’s Hospital, School of Medicine, Tongji University, Shanghai 200072, P. R. China; Center for Orthopaedic Science and Translational Medicine, Department of Orthopedics, Shanghai Tenth People’s Hospital, School of Medicine, Tongji University, Shanghai 200072, P. R. China; Department of Orthopedics, Changzheng Hospital, Second Military Medical University (Naval Medical University), Shanghai 200003, P. R. China; Center for Orthopaedic Science and Translational Medicine, Department of Orthopedics, Shanghai Tenth People’s Hospital, School of Medicine, Tongji University, Shanghai 200072, P. R. China; Center for Orthopaedic Science and Translational Medicine, Department of Orthopedics, Shanghai Tenth People’s Hospital, School of Medicine, Tongji University, Shanghai 200072, P. R. China; Center for Orthopaedic Science and Translational Medicine, Department of Orthopedics, Shanghai Tenth People’s Hospital, School of Medicine, Tongji University, Shanghai 200072, P. R. China; Center for Orthopaedic Science and Translational Medicine, Department of Orthopedics, Shanghai Tenth People’s Hospital, School of Medicine, Tongji University, Shanghai 200072, P. R. China; Institute of General and Inorganic Chemistry, National Academy of Sciences of Belarus, Minsk 220072, Belarus; Institute of General and Inorganic Chemistry, National Academy of Sciences of Belarus, Minsk 220072, Belarus; Center for Orthopaedic Science and Translational Medicine, Department of Orthopedics, Shanghai Tenth People’s Hospital, School of Medicine, Tongji University, Shanghai 200072, P. R. China; Center for Orthopaedic Science and Translational Medicine, Department of Orthopedics, Shanghai Tenth People’s Hospital, School of Medicine, Tongji University, Shanghai 200072, P. R. China; Center for Orthopaedic Science and Translational Medicine, Department of Orthopedics, Shanghai Tenth People’s Hospital, School of Medicine, Tongji University, Shanghai 200072, P. R. China; Institute of General and Inorganic Chemistry, National Academy of Sciences of Belarus, Minsk 220072, Belarus; Center for Orthopaedic Science and Translational Medicine, Department of Orthopedics, Shanghai Tenth People’s Hospital, School of Medicine, Tongji University, Shanghai 200072, P. R. China; Suzhou First People’s Hospital, School of Medicine, Anhui University of Science and Technology, Anhui 232001, P. R. China

**Keywords:** magnesium phosphate cement, bone repair, bone adhesive, strong adhesion, screw fixation

## Abstract

Conventional bone cements face critical limitations in biodegradability, bioactivity and mechanical compatibility. To overcome these challenges, an all-in-one injectable magnesium phosphate cement (MPC) is engineered for bone repair, fracture adhesion and osteoporotic fixation. This functional platform integrates rapid self-setting, high early compressive strength and controlled degradation synchronized with bone formation. Unlike bioinert poly(methyl methacrylate) or slow-degrading calcium phosphate cements, MPC offers a superior bioactive alternative. It promotes bone regeneration through the sustained release of osteogenic Mg^2+^ ions, which accelerate osteoblast differentiation and angiogenesis. Comprehensive characterization confirms MPC’s dense microstructure, mild exothermic reaction, physiological pH stability and biocompatible degradation, eliminating risks of thermal necrosis or toxic ion accumulation. The MPC demonstrates outstanding initial mechanical properties under *in vivo*-like conditions characterized by a warm and humid environment. *In vitro* studies show that MPC significantly promotes cell migration, upregulates the expression of osteogenic markers and enhances mineralized matrix deposition. Its controlled degradation behavior sustainably releases osteogenic Mg^2+^ ions, which orchestrate the proliferation of bone marrow stromal cells and facilitate RUNX2-mediated osteogenic differentiation, collectively accelerating the mineralization process. *In vivo* evaluations further reveal multi-functional bone regenerative capabilities: MPC dynamically guides defect repair through degradation-coupled bone ingrowth, achieving seamless integration without interfacial gaps, and significantly augments screw fixation stability via robust osseointegration. With exceptional adhesion to diverse substrates (tantalum, PLA, bone; 5.5× stronger than CPC on PLA) and intrinsic safety, MPC establishes an advanced platform for orthopedic regeneration and fixation.

## Graphical abstract

**Figure:**
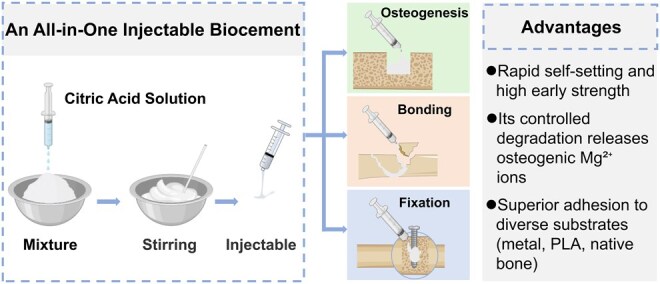

## Introduction

The rising incidence of bone defects, driven by an aging global population, traumatic injuries and pathological conditions like osteoporosis and periodontitis, has intensified demand for advanced orthopedic biomaterials [1, 2]. While minor bone injuries may self-heal, critical-sized defects often surpass innate regenerative capacity, imposing significant clinical and socioeconomic burdens [3–6]. Current gold-standard autografts face limitations including donor scarcity, morbidity and infection risks, prompting exploration of synthetic alternatives [7]. Metallic implants, such as porous titanium alloys, provide essential structural integrity but often lack inherent bioactivity [8, 9]. Polymeric scaffolds, including materials like PCL and PLA, offer the advantage of tunable degradation profiles, yet frequently demonstrate insufficient osteoconductive properties [10, 11]. Bioceramics, exemplified by hydroxyapatite, closely mimic the mineral composition of bone but are typically limited by their inherent brittleness [12–16]. Given these limitations associated with current synthetic bone substitutes, injectable bone cements have become a clinically indispensable solution. They uniquely fulfill the critical need for immediate mechanical stabilization while enabling precise, defect-conformal filling.

Injectable bone cements are broadly classified into organic and inorganic variants. The organic cements typified by polymethylmethacrylate (PMMA) delivering high compressive strength (70–100 MPa) and rapid polymerization, yet plagued by exothermic reactions (>80°C causing thermal necrosis), bioinertness, non-degradability, monomer-induced cytotoxicity and risks of aseptic loosening or chronic inflammation [17–20]. The inorganic cements represented by calcium phosphate cements (CPCs), which offer intrinsic biodegradability and osteoconductivity but suffer from low initial mechanical strength (5–40 MPa), degradation–regeneration mismatch, poor physiological washout resistance and prolonged setting kinetics (∼30 min) incompatible with intraoperative handling requirements [21–25]. Magnesium phosphate cements (MPCs) emerge as transformative bioactive alternatives addressing these limitations [26]. Comprising MgO and phosphate salts (e.g. KH_2_PO_4_), MPCs uniquely combine rapid setting (5–15 min) with high early strength (20–50 MPa) for immediate load-bearing [14, 27–29] alongside controllable degradation (3–10 months) synchronized with bone regeneration [30]. Critically, their degradation releases osteogenic Mg^2+^, as the fourth most abundant physiological cation involved in >300 enzymatic reactions, which activates PI3K/Akt signaling to stimulate osteoblast proliferation, RUNX2-mediated osteogenic differentiation, angiogenesis and nerve regeneration [31–35]. This intrinsic bioactivity enables dynamic degradation-guided bone formation, circumventing repair failure risks inherent to traditional material–bone regeneration mismatches [36, 37]. Moreover, the treatment of comminuted fractures and complex bone defects represents a formidable clinical challenge in orthopedic practice, creating an imperative demand for tailored repair materials. Conventional metallic fixation devices (e.g. plates/screws) frequently fail to stabilize morphologically intricate fragments, leading to delayed union or nonunion [38–40]. The bone–implant interface remains susceptible to mechanical loosening particularly in osteoporotic bone, due to stress shielding and insufficient osseointegration [41–43]. Injectable bone cements offer a promising strategy to simultaneously augment screw fixation and bond fracture fragments. Yet traditional PMMA cements suffer from non-degradability, monomer toxicity and exothermic polymerization, compromising long-term stability and impeding bone regeneration [18, 19]. While biodegradable CPCs address degradation concerns, their brittleness, slow setting and poor washout resistance limit utility in load-bearing scenarios [13, 25].

**Figure 1:**
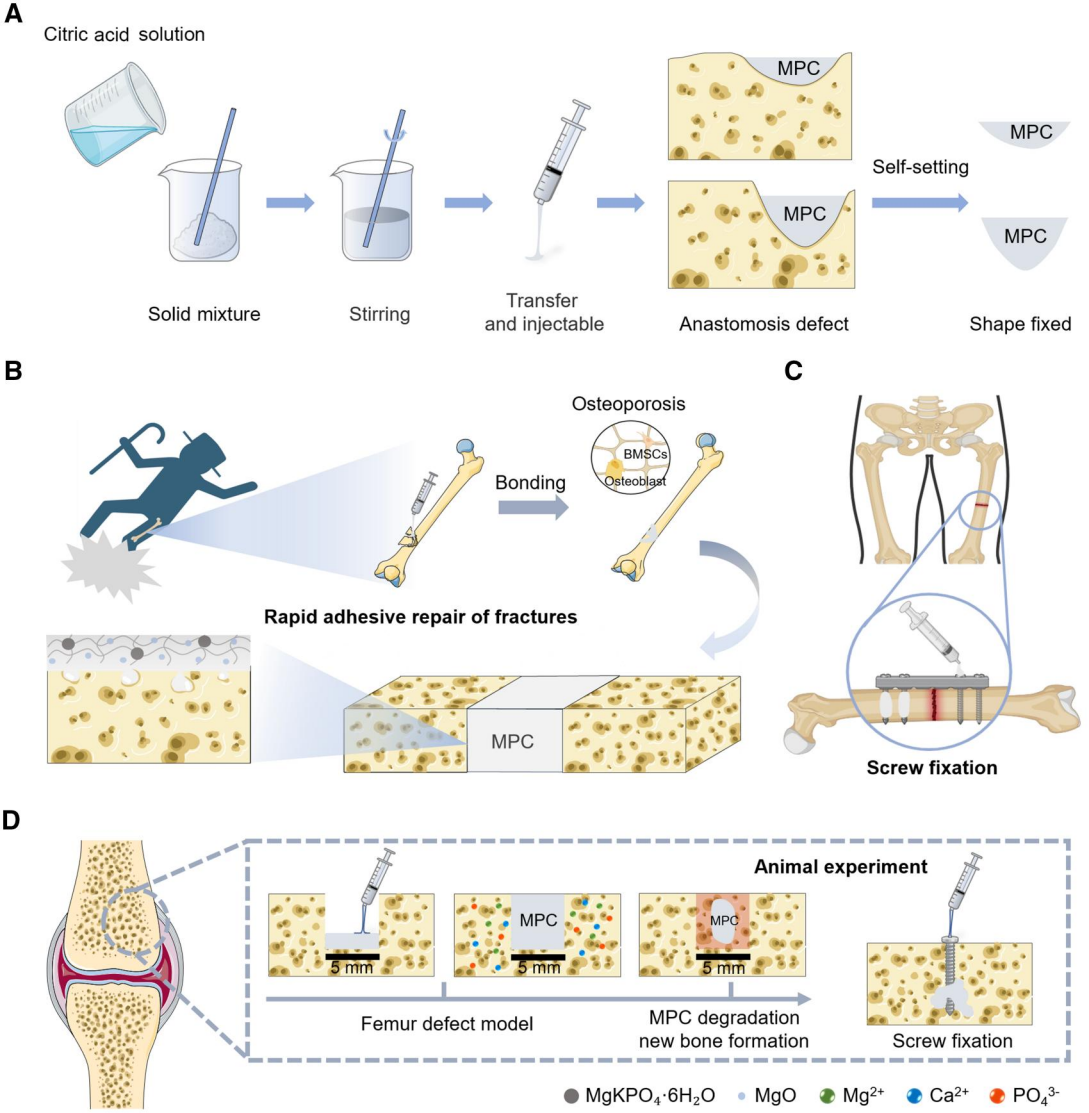
Schematic diagram of the preparation strategy for degradable bioactive MPC, as well as its role in promoting osteogenesis at the femoral defect site, facilitating rapid bonding of comminuted bone defects and achieving screw fixation.

Although MPCs have been extensively investigated, existing studies have largely focused on optimizing individual properties. Distinct from previous studies, this work achieves, for the first time, the multi-functional integration of rapid bone repair, fracture adhesion and implant augmentation within a single MPC system. Based on this design, we aim to develop a highly bioactive and degradable MPC platform engineered as an all-in-one injectable biocement (Figure 1). The term ‘all-in-one’ denotes a unified material system that combines bone repair, fracture adhesion and osteoporotic fixation within one injectable and self-setting MPC. By optimizing formulations based on magnesium phosphate and potassium dihydrogen phosphate systems, we simultaneously enhance mechanical functionality and leverage sustained Mg^2+^ release to activate osteogenic gene expression (e.g. OCN, RUNX2), accelerating bone marrow mesenchymal stem cell differentiation into osteoblasts. This establishes a degradation-guided bone regeneration paradigm that dynamically synchronizes material resorption with new bone formation. Crucially, this singular MPC platform delivers a therapeutic material with multiple functions, providing structural defect repair, fracture adhesion and enabling biological implant fixation through superior osseointegration. This integrated approach uniquely positions MPC to address critical challenges, by offering robust adhesion for comminuted fractures and targeted reinforcement in osteoporotic bone, thereby potentially reducing postoperative complications and accelerating the clinical translation of MPCs.

## Materials and methods

### Material preparation

Firstly, KH_2_PO_4_, which had been dried at 150°C for 20 h, was ground in a planetary ball mill at a speed of 300 rpm for 1 h and then passed through a 200-mesh sieve for later use. Mg(OH)_2_ was calcined at 1300°C for 6 h in a muffle furnace to obtain MgO. The heating rate of the furnace was 10°C/min. After cooling, the MgO was passed through a 200-mesh sieve and reserved. Mg_3_(PO_4_)_2_ and Ca(H_2_PO_4_)_2_ were also passed through a 200-mesh sieve and kept for later use. Sucrose was ground in a planetary ball mill at a speed of 300 rpm for 1 h and then passed through a 200-mesh sieve for later use. The prepared KH_2_PO_4_, MgO, Mg_3_(PO_4_)_2_, Ca(H_2_PO_4_)_2_ and sucrose were mixed uniformly and then irradiated together with a 0.03 g/mL citric acid solution as the liquid phase to complete the preparation of the bone cement raw materials.

The irradiated solid powder was placed in a beaker, and the irradiated liquid phase was added. After stirring for 3 min, the mixture was transferred to a syringe and cast into a corresponding mold. After setting for 3 h, the sample was demolded and then hydrated in a 100% humidity environment for 24 h to obtain the target bone cement.

### Setting property determination

The irradiated solid powder was placed in a mixing cup, and the irradiated liquid phase was added. After stirring for 3 min, the mixture was transferred to a syringe and injected into a 7-mL centrifuge tube. Subsequently, a temperature probe was inserted into the bone cement, and the temperature was measured every 30 s until the peak temperature was reached, at which point the test was stopped. Another batch of irradiated solid and liquid powders was taken, stirred for 3 min, transferred to a syringe and injected into a 7-mL centrifuge tube. A viscosity probe was inserted into the bone cement, and the viscosity was measured every 30 s until it exceeded the maximum range of the instrument (DV3T rheometer).

### Microstructure of the material

The external and internal microstructures of the bone cement samples after 24 h of hydration were observed and photographed using a scanning electron microscope (SEM; ZEISS GeminiSEM 300). The elemental composition of the samples was also analyzed using the EDS (Energy Dispersive Spectroscopy) function of the SEM.

### Short-term pH of the MPC

The irradiated solid powder was placed in a beaker, and the irradiated liquid phase was added. After stirring for 3 min, deionized water was slowly added at a mass ratio of 0.2 g/mL. The mixture was then placed in a water bath at 37°C, with the pH probe fully immersed in the deionized water. The pH was measured every 30 s for a continuous period of 24 h (pH meter: PXSJ-216F).

### Long-term pH of the MPC

The bone cement samples, which had been hydrated for 24 h and shaped into cylindrical specimens (Φ5 × 5 mm), were naturally dried. They were then immersed in PBS at a solid-to-liquid ratio of 0.2 g/mL. The centrifuge tubes were capped and placed in a shaker at 37°C with a shaking frequency of 100 rpm. The supernatant pH was measured at predetermined times.

### Anti-disintegration test

The irradiated solid powder was placed in a mixing cup, and the irradiated liquid phase was added. After stirring for 3 min, the mixture was transferred to a syringe and injected into a 50-mL centrifuge tube filled with deionized water. The tube was capped and placed in a shaker at 37°C with a shaking frequency of 60 rpm for 24 h. The supernatant and two rinsing liquids were then collected in a beaker and dried in an oven. The weight difference between the beaker and the dried sample represented the disintegrated portion of the bone cement during injection.

### *In vitro* degradation

The irradiated solid powder was placed in a beaker, and the irradiated liquid phase was added. After stirring for 1.5 min, the mixture was transferred to a syringe and cast into cylindrical molds (Φ5 × 5 mm). After demolding at 3 h, the initial mass of the bone cement was measured. The cylindrical bone cement samples were then placed in centrifuge tubes containing PBS at a ratio of 0.015 g/mL and incubated in an oven at 37°C with a rotation speed of 100 rpm. The buffer solution was changed every 2 days. At predetermined times, the samples were removed, dried in an oven at 60°C for 4 h and their masses were measured to calculate the weight loss. The samples were then returned to the centrifuge tubes for further soaking until the next measurement.

### XRD analysis

Bone cement samples hydrated for 24 h were ground into powder and sent for XRD testing (Empyrean, Malvern Panalytical, Netherlands). The samples were scanned using a Cu target over a range of 5°–90° at a rate of 10°/min.

### Effect of irradiation on compressive strength

The physical forms of the solid and liquid phases before and after irradiation were observed. The compressive strength of bone cement samples prepared from different treatment groups was compared. The equipment used for irradiation was the IS1020—linear electron accelerator. The energy of the electron beam of the equipment was 10 MeV, the power was 20 kW and the irradiation dose was 25 kGy. The irradiated powder–liquid group consisted of irradiated solid powder and irradiated citric acid liquid phase; the single-irradiated powder group consisted of irradiated solid powder and non-irradiated citric acid liquid phase; the single-irradiated liquid group consisted of non-irradiated solid powder and irradiated citric acid liquid phase. The liquid used for the contrast photography, which had undergone sterilization treatment under high temperature and high pressure, was sterilized at 121°C for 15 min. The test samples were cylindrical specimens (Φ5 × 5 mm) that were hydrated in a 37°C, 100% humidity environment for 24 h. The compressive strength was measured using a universal testing machine with a 200-kg load cell and a crosshead speed of 3 mm/min. The ends of the bone cement samples were polished with 300–400 mesh sandpaper before testing. Each group consisted of at least three parallel samples.

### Compressive strength over setting time

The irradiated solid powder was placed in a beaker, and the irradiated liquid phase was added. After stirring for 3 min, the mixture was transferred to a syringe and cast into cylindrical molds (Φ5 × 5 mm). After demolding at 3 h, the samples were cured in a 37°C, 100% humidity environment for different durations. The compressive strength of the hydrated bone cement was measured using a universal testing machine with a 200-kg load cell and a crosshead speed of 3 mm/min. The ends of the bone cement samples were polished with 300–400 mesh sandpaper before testing. Each group consisted of at least three parallel samples.

### Compressive strength over soaking time

The irradiated solid powder was placed in a beaker, and the irradiated liquid phase was added. After stirring for 3 min, the mixture was transferred to a syringe and cast into cylindrical molds (Φ5 × 5 mm). After demolding at 3 h, the samples were naturally dried and then placed in centrifuge tubes containing PBS at a ratio of 0.2 g/mL. The tubes were placed in an oven at 37°C with a rotation speed of 100 rpm. At predetermined times, the samples were removed, dried in a universal testing machine and their compressive strengths were measured. The tests were conducted using a 200-kg load cell and a crosshead speed of 1 mm/s. The ends of the bone cement samples were polished with 300–400 mesh sandpaper before testing. Each group consisted of at least three parallel samples.

### Accelerated aging experiment of MPC liquid phase

According to the provisions of YY/T0681.1—Test Methods for Sterile Medical Device Packaging—Part 1: Accelerated Aging Test Guidelines, an accelerated aging test model was established (with a humidity of 60%).


$$\mathrm{AAF}=Q_{10}^{\left[(T_{\mathrm{AA}}-T_{\mathrm{RT}})/10\right]}$$

Among them, *Q*_10_ was selected as 2, *T*_AA_ was the accelerated aging temperature, which was set at 60°C, and *T*_RT_ was the actual storage temperature, which was set at 25°C. The Accelerated Aging Factor (AAF) is a mathematical parameter used to determine how much faster a product ages at an elevated temperature compared to normal ambient storage. After calculation, AAF ≈ 11.37


$$\mathrm{AAT}={\mathrm{RT}}_{Y}/\mathrm{AAF}$$

RT_Y_: the expected aging time is set at 6 months, which is equivalent to 183 days. After calculation, therefore, AAT = 183 days/11.37 ≈ 16 days.

Therefore, under the conditions of a storage temperature of 60°C and a humidity of 60%, for 16 days, it is equivalent to being stored at 25°C for 183 days. The validity period is 6 months. The same applies to 1 month and 3 months.

### *In vitro* screw pull-out test

Cancellous bone models were cut into rectangular prisms (5 cm × 5 cm × 4 cm), and orthopedic fixation screws were drilled into them. The blank group had screws without any bone cement reinforcement; the PMMA group had screws reinforced with commercial PMMA bone cement; the MPC group had screws reinforced with the optimized MPC. After mixing for 3 min, the bone cement was injected into the screws using a plunger, with 2 mL of cement per screw. The samples were hydrated in a 37°C, 100% humidity environment for different durations. The maximum pull-out force of the screws was measured using a universal testing machine with a 200-kg load cell and an upward speed of 10 mm/s. Each group consisted of at least three parallel samples.

### *In vitro* adhesion performance test

The bovine bone and cancellous bone models were sectioned and prepared for use. After mixing MPC for 3 min, it was extruded via syringe onto the cross-section of the bovine bone/cancellous bone model. The samples were hydrated at 37°C in 100% humidity for 24 h, then subjected to a 2 kg hanging weight to observe their load-bearing capacity.

Bovine bone was immersed in hydrogen peroxide solution for 3 days for defatting, then cut into blocks. PLA scaffolds were trimmed to appropriate dimensions. Tantalum metal porous scaffolds were prepared as well. The MPC was mixed for 3 min and extruded via syringe onto the bonding surfaces of the blocks. The opposing surfaces were gently pressed together for adhesion. The bonded specimens were hydrated at 37°C in 100% humidity for 24 h. Following hydration, the samples were removed and tested using a universal testing machine with a 200-kg load cell and an upward speed of 10 mm/s to measure the maximum pull-out force at the bonding interface. Each group consisted of at least three parallel samples.

### *In vitro* cell viability test

Magnesium phosphate bone cement samples hydrated for 24 h were sterilized using autoclaving and placed in 50-mL centrifuge tubes. An extraction medium consisting of α-MEM culture medium with 10% fetal bovine serum was added at a ratio of 0.2 g/mL. The samples were incubated in a constant temperature shaker at 37°C and 60 rpm for 24 h to obtain the extraction liquid of the magnesium phosphate bone cement.

In a 96-well plate, 1 × 10^4^ bone marrow stromal cells (BMSCs) were added to each well and cultured overnight at 37°C in a 5% CO_2_ humidified incubator to allow cell attachment. After 24 h, the cells were rinsed with PBS buffer (pH = 7.4) and then co-cultured with different concentrations of material extract (0%, 12.5%, 25%, 50%, 100%) at 37°C to assess the cytotoxicity of the material. After 24 h of co-culture, 10 μL of 5 mg/mL MTT reagent was added to each well. After incubation at 37°C for 4 h, the supernatant was removed, and 150 μL of dimethyl sulfoxide was added and shaken for 10 min. The absorbance of the supernatant at 490 nm was measured using a microplate reader. Cell viability (%) = (OD of cells with material/OD of control cells) × 100%. Additionally, the cytotoxicity of the material extract after 24 and 48 h of co-culture with cells was assessed using a Calcein/PI cell viability and dead cell staining kit (Beyotime).

In a 24-well plate, 4 × 10^4^ BMSCs were added to each well. After 24 h, 500 μL of material extract was added. After 24 h of treatment, cell cytoskeleton staining was performed using a microtubule green fluorescence staining kit (Beyotime), and cell morphology and activity were observed under bright-field and laser excitation conditions. Cells co-cultured with cylindrical magnesium phosphate bone cement (Φ10 × 2.5 mm) for 24 h were fixed with paraformaldehyde and stained with calcein or directly observed under a scanning electron microscope (SEM) to evaluate the interaction between cells and the material. When the cells reached 80% confluence at the bottom of the well, the culture medium was replaced with osteogenic induction medium and continued to be cultured until day 7. The alkaline phosphatase (ALP) activity was measured using a BCIP/NBT ALP staining kit (Beyotime). On day 14, alizarin red staining solution (OriCell) was used to observe the calcified nodules of the cells.

### Quantitative PCR experiment

In a 6-well plate, 5 × 10^4^ BMSCs were added to each well. After 24 h, 2 mL of material extract was added to each well, and the extract medium was changed every 3 days. After 7 days, 1 mL of Trizol was added to each well to extract RNA. Reverse transcription was performed using the PrimeScript™ RT reagent Kit with gDNA Eraser (Perfect Real Time) from TaKaRa. Quantitative PCR was performed using the TB Green^®^ Premix Ex Taq™ II (Tli RNaseH Plus) kit from TaKaRa. The primers used are listed in Supplementary Table S2.

### Western blot experiment

In a 6-well plate, 3 × 10^6^ BMSCs were added to each well. After 24 h, 2 mL of material extract was added to each well. The extract medium was changed every 3 days. When the cells reached 80% confluence at the bottom of the well, the medium was replaced with osteogenic induction medium containing the material extract. On days 7 and 14, cells were collected for Western blot analysis. The cells were lysed in 1.5-mL EP tubes on ice, and the sample concentration was adjusted to 5 μg/μL. A total of 10 μL of each sample was loaded per well. After the blue band reached the appropriate position, the membrane was transferred. After transfer, the NC membrane was trimmed as needed and blocked with 7% milk (dissolved in 1×PBS). After blocking, primary antibodies were added: anti-osteocalcin (1:1000, Santa Cruz, USA), anti-actin (1:1000, Abcam, UK) and incubated overnight at 4°C. After three washes, secondary antibody anti-mouse second antibody (1:3000, CST, USA) was added and incubated for 1 h at room temperature on a rocker in the dark. The NC membrane was scanned using an infrared laser scanner.

### *In vivo* osteogenesis induction of bone cement

The osteoinductive properties of the bone cement were explored using a femoral defect model in New Zealand white rabbits. This study was approved by the Ethics Committee of Shanghai Tenth People’s Hospital Affiliated to Tongji University (Ethics No.: SHDSYY-2023-66200412). New Zealand white rabbits were purchased from Shanghai Jiagan Biotechnology Co., Ltd, with 15 males and 15 females, 6 months old and in good health. The bone defect experiment was divided into three groups. Thirty experimental rabbits were marked using the separate cage method and randomly divided into a blank group, a control group (implanted with commercial CPC, and calcium sulfate cement, CSC) and an experimental group (MPC), with 12 rabbits in the blank and control groups and 12 in the experimental group, and six rabbits kept as backups. A cylindrical defect with a diameter of 6 mm and a height of 10 mm was created at the distal end of the rabbit femur, and each group’s bone cement was filled into the defect. After surgery, at predetermined times, ear vein blood was collected from rabbits in the MPC group and sent to YouBio for rabbit blood, biochemical and electrolyte tests. Rabbit femurs implanted for 1 month, 3 months and 6 months were sent to Compass for 3D reconstruction and data analysis of new bone tissue. The rabbit femurs that had undergone Micro-CT were sent to Sever for decalcification of bone tissue, followed by hematoxylin–eosin (HE) staining and Masson’s trichrome staining.

### *In vivo* screw reinforcement pull-out

The screw reinforcement model involved drilling a hole parallel to the joint surface at a depth of 10 mm and a distance of 4 mm from the distal end of the rabbit femoral joint surface using a solid drill with an inner diameter of 6 mm. In one leg of the rabbit, bone cement was filled into the hole, and a φ7 × 10 mm metal bone screw was inserted into the bone channel; in the other leg, no bone cement was filled, and the screw was inserted. The tail of the screw was parallel to the bone interface to ensure consistent implantation depth. New Zealand white rabbits were purchased from Shanghai Jiagan Biotechnology Co., Ltd, with 12 males and 12 females, 6 months old, weighing approximately 2.5–3.0 kg and in good health. The screw reinforcement fixation was divided into two groups. Twenty-four experimental rabbits were marked using the separate cage method and randomly divided into a blank control group and a screw reinforcement fixation group, with 12 rabbits in each group. After surgery, the rabbits were raised until the designated time, at which point the entire femur was removed and tested on a universal testing machine to measure the force required to pull-out the screw.

### Statistical analysis

Data were analyzed using GraphPad Prism 8.0 (GraphPad Software, La Jolla, CA, USA) and SPSS statistical software version 26 (IBM, Armonk, NY). All data are presented as mean ± standard deviation (SD). Differences between two or multiple groups were assessed using *t*-tests and one-way or two-way analysis of variance (ANOVA). Significance levels were set at *P < 0.05, **P < 0.01, ***P < 0.001, and ****P < 0.0001.

## Results and discussion

### The chemical reaction and solidification of MPC

MPC is an inorganic material comprising calcined MgO, soluble phosphates, retarders and minerals. When mixed with water under acidic conditions, it undergoes acid–base reactions and physical interactions to form a phosphate-bound matrix [29]. Hydration products include struvite (MgNH_4_PO_4_·6H_2_O) or similar magnesium phosphate compounds. However, ammonia release during MgO/ammonium dihydrogen phosphate hydration poses equipment and health risks. To address this, Wagh developed magnesium potassium phosphate cement (MKPC) by substituting potassium phosphate for ammonium phosphate, with MgKPO_4_·6H_2_O (MKP) as the primary hydration product [44–49]. The MKPC hydration process involves five stages, including dissolution of phosphate and ionization of MgO, hydration of MgO to form aqueous Mg^2+^ ions, reaction of Mg^2+^ with phosphate ions to form magnesium phosphate gel, gel interconnection and crystallization into a network structure [44, 50].

Upon mixing MPC’s solid and liquid phases, potassium dihydrogen phosphate dissolution acidifies the solution. MgO and Mg_3_(PO_4_)_2_ subsequently undergo hydrolysis, releasing Mg^2+^ ions. When Mg^2+^ and dihydrogen phosphate concentrations reach critical levels, MKP precipitation initiates. Larger MgO specific surface areas accelerate MKP formation and increase yield. Hydration products accumulate progressively, forming a gel phase where crystal nuclei interconnect and densify. Unreacted MgO particles provide structural support, while hydration products fill interstitial spaces, creating a cohesive 3D crystalline network. This microstructural integration confers exceptional mechanical strength to the cured material.

### Physicochemical characterization of MPC

**Figure 2:**
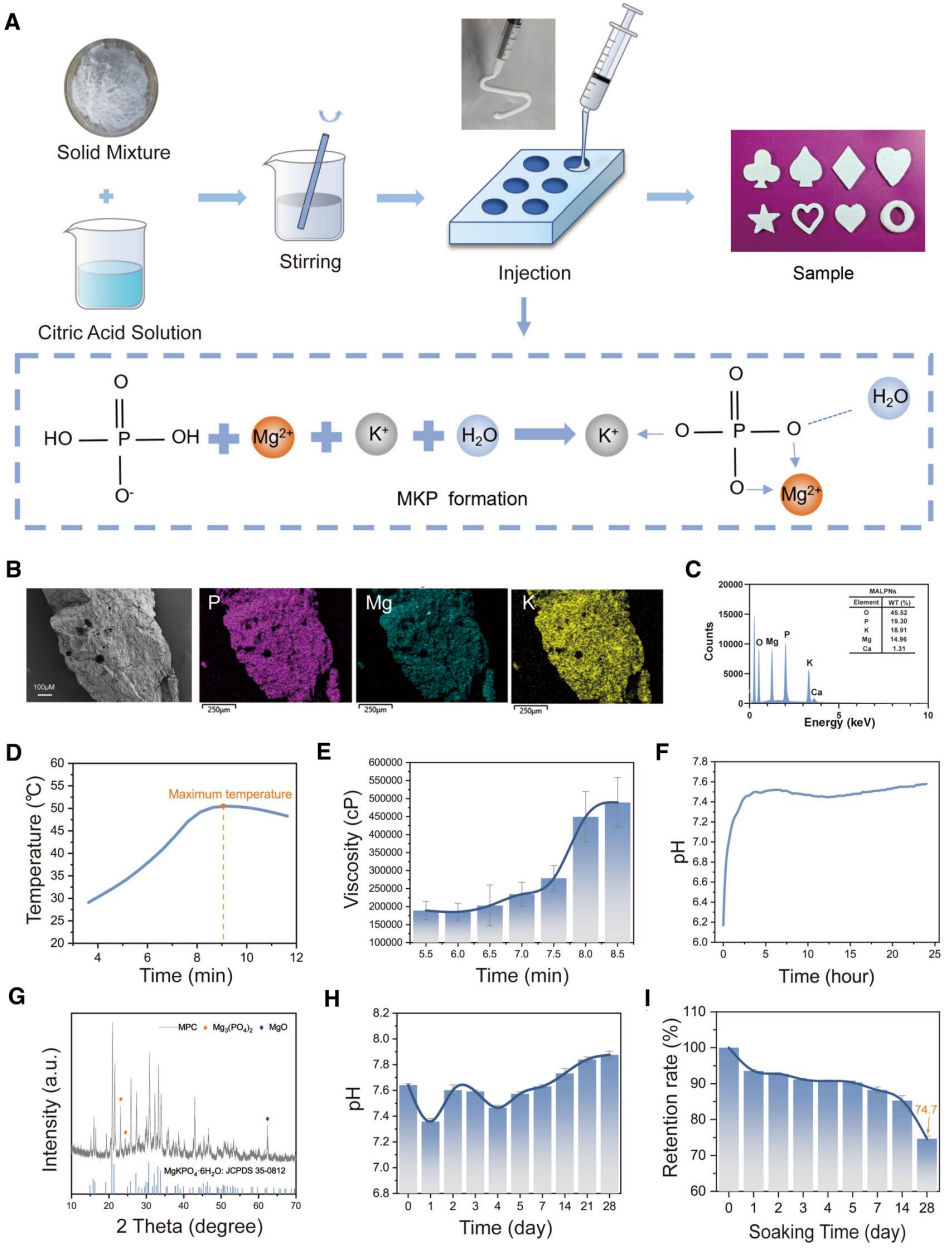
Fabrication and physicochemical characterization of magnesium phosphate cement (MPC). (**A**) Specimen fabrication protocol and the reaction mechanism of MPC. (**B**–**C**) Cross-sectional SEM morphology showing limited microporosity (**B**) and EDS elemental mapping (**C**). (**D**–**F**) Reaction kinetics: exothermic profile peaking at 51°C (**D**), viscosity evolution (**E**) and pH trajectory (**F**) during setting (*n* = 3). (**G**) XRD pattern identifying MgKPO_4_·6H_2_O (JCPDS 35-0812) as the primary crystalline phase, with minor MgO (JCPDS 45-0946) and Mg_3_(PO_4_)_2_. (**H**) pH stability in PBS over 28 days (*n* = 3). (**I**) *In vitro* degradation profile (*n* = 3).

Through systematic optimization of raw material processing, premixing protocols and formulation parameters, we successfully fabricated MPC to meet comprehensive clinical requirements. Cylindrical specimens (*Φ*5 × 5 mm) were molded for physicochemical characterization (Figure 2A). Phase composition analysis via XRD identified potassium magnesium phosphate hexahydrate (MgKPO_4_·6H_2_O, JCPDS No. 35-0812) as the dominant crystalline phase, with minor peaks corresponding to unreacted MgO (periclase, JCPDS No. 45-0946) and residual Mg_3_(PO_4_)_2_ (Figure 2G). SEM analysis confirmed the dense microstructure of as-prepared MPC cylinders, with only sparse microporosity (pore diameter ≤100 μm) observable internally (Figure 2B). This limited porosity likely originated from gas entrapment during sample preparation and cement setting processes. Mercury intrusion porosimetry (MIP) quantified a nanoporous architecture with an average pore diameter of 157.2 nm and ultralow porosity (4.10%), further validating the predominantly compact structure observed by SEM (Table S1). Externally, MPC exhibited a coherent surface composed of tightly packed crystallites (0.5–5 μm) without compromising structural integrity (Supplementary Figure S2). Minor cracks observed on cross-sections represented processing artifacts rather than material defects. Elemental mapping by EDS confirmed homogeneous distribution of Ca, K, P, Mg and O throughout the matrix (Figure 2B and C), consistent with XRD quantification.

The physical changes occurring during the cement setting process were systematically monitored. For the optimized formulation, the temperature steadily increased throughout the reaction, reaching a peak of 51°C at 9 min before gradually declining (Figure 2D). This indicates a mild exothermic reaction, with the peak temperature significantly lower than that of PMMA bone cement, thereby eliminating the risk of thermal damage to surrounding tissues upon implantation. Following the mixing of solid and liquid phases, viscosity initially decreased with stirring. Stirring was halted at 3 min, and the cement was transferred for rheological testing. Viscosity then progressively increased during the reaction, exceeding the rheometer’s measurement range at approximately 8 min—coinciding with the peak exothermic temperature. This confirms substantial heat release during the setting process (Figure 2E).

pH monitoring revealed an initial acidic environment, attributed to unreacted citric acid. As the reaction progressed, the pH rose rapidly, likely due to acid–base neutralization, peaking at around 5 h before declining (Figure 2F). This subsequent decline may result from the precipitation of ions (potassium, magnesium), involving the hydration of MgO to form Mg(OH)_2_. However, continued Mg^2+^ release led to a gradual pH increase after 12 h, stabilizing below 7.6. When immersed in PBS solution, the MPC’s pH initially decreased due to the dissolution of acidic components but stabilized within the weakly alkaline range (7.3–7.7) over time (Figure 2H). This stabilization suggests a controlled release of Mg^2+^ ions, ensuring long-term biocompatibility without local pH-induced tissue damage. Controlled degradation of the MPC *in vitro* was evidenced by weight loss rates of 15% at 14 days and 25% at 28 days (Figure 2I). This degradation profile enables the sustained release of osteogenic ions (Ca^2+^/Mg^2+^), facilitating synchronization between material resorption and new bone formation. Considering the potential risks associated with chronic heavy metal release from implants, ICP analysis confirmed that the MPC’s heavy metal content was well below 5 ppm (Supplementary Figure S3), fully compliant with safety standards for medical implants. These collective physicochemical assessments highlight the MPC’s suitability as a clinically viable, bioactive and degradable bone repair material with optimized properties.

### Mechanical properties of MPC

**Figure 3:**
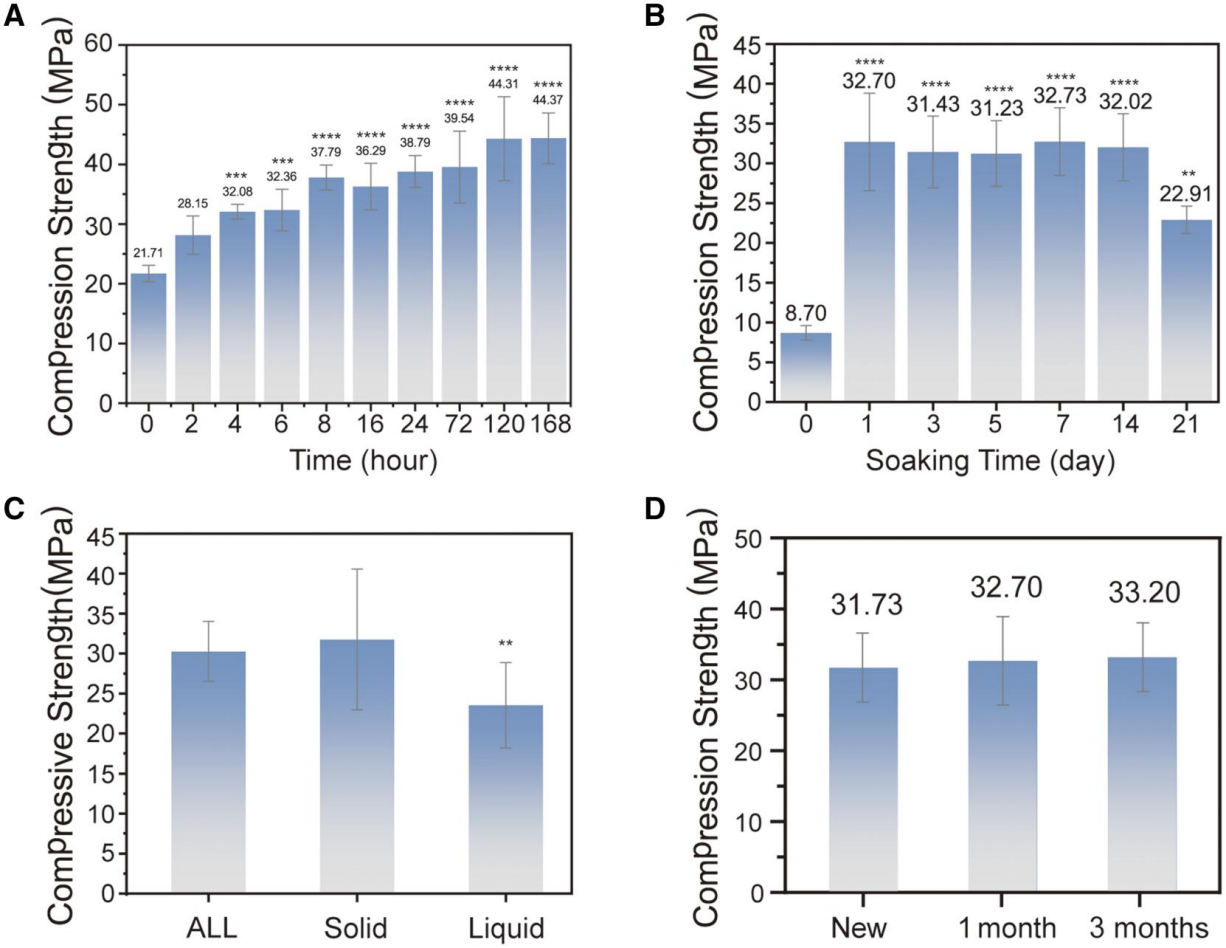
*In vitro* mechanical properties of MPC. (**A**) Compressive strength versus setting time (*n* = 6). (**B**) Compressive strength after immersion in PBS for varying durations (*n* = 6). (**C**) Compressive strength after dual-phase irradiation (solid + liquid), solid-phase-only irradiation, or liquid-phase-only irradiation (*n* = 6). (**D**) Compressive strength after different storage times of citric acid solution (*n* = 6). Data presented as mean ± SD; **P < 0.01, ***P < 0.001, ****P < 0.0001.

The mechanical performance of MPC, as a novel bioactive bone repair material, critically governs its clinical applicability, safety and long-term efficacy. Analysis of its mechanical properties revealed that MPC’s compressive strength increased progressively with hydration time. After 4 h of hydration, the average compressive strength exceeded 30 MPa, stabilized above 35 MPa after 8 h and surpassed 40 MPa following 5 days of setting, demonstrating excellent mechanical performance for this formulation (Figure 3A). To evaluate performance in clinically relevant scenarios, the effect of immersion duration on compressive strength was further investigated. Post-immersion, MPC’s compressive strength significantly increased, reaching 32.7 MPa at day 1. The average compressive strength remained stable at approximately 32 MPa within the first 14 days of immersion, with no substantial decline. However, after 21 days of immersion, the average strength decreased to 22.91 MPa (Figure 3B). Incorporating sucrose into the formulation did not affect compressive strength but prolonged the reaction time. Conversely, omitting sucrose drastically shortened the reaction time, causing rapid solidification of MPC, which is undesirable for clinical handling (Supplementary Figure S6).

Exposure to radiation, a common method of commercial sterilization, can cause physical changes in materials that impair their performance [51]. The irradiation induces structural changes in the precursors rather than in the forming MPCs, since phosphate cements, as a rule, retain their strength under the influence of gamma irradiation [52, 53]. At the same time, the effect of gamma irradiation on MgO with a dose of 20 kGy contributes to an increase in the size of its crystallites from 11 to 16 nm [54], which can slow down the rate of interaction with KH_2_PO_4_ and the strength gain of the forming MPC. As for the liquid phase, the effect of gamma radiation contributes to the destruction of the molecules of the complexing agent, citric acid—the degree of radiolysis reaches 40% upon absorption by a 5 mM acid solution at a dose of 25 kGy [55], as a result of which the introduction of the complexing agent does not ensure an increase in the strength of the MPC. It is noteworthy that this effect is observed only when an irradiated liquid phase is introduced into the composition of the MPC, since when only the solid or solid and liquid phases are irradiated, the resulting MPCs are characterized by comparable strength. To address clinical requirements, we investigated the mechanical effects of irradiating MPC’s solid and liquid phases. Post-irradiation comparison revealed that irradiation caused yellowing of the liquid phase when both sucrose and citric acid were present (Supplementary Figure S7). Irradiation of both solid and liquid phases reduced MPC’s strength to 30 MPa, while irradiation of the liquid phase alone exerted a more pronounced negative impact on compressive strength (Figure 3C). To meet industrial production needs, the long-term stability of MPC’s solid and liquid phases was evaluated. Long-term storage of citric acid solution resulted in MPC thinning during mixing and shortened setting time, but did not compromise compressive strength (Figure 3D). Accelerated aging experiments (citric acid solution stored at 60°C for 16 days, simulating 6 months of storage) confirmed that the stored solution did not reduce the cement’s compressive strength (Supplementary Figure S8).

**Figure 4:**
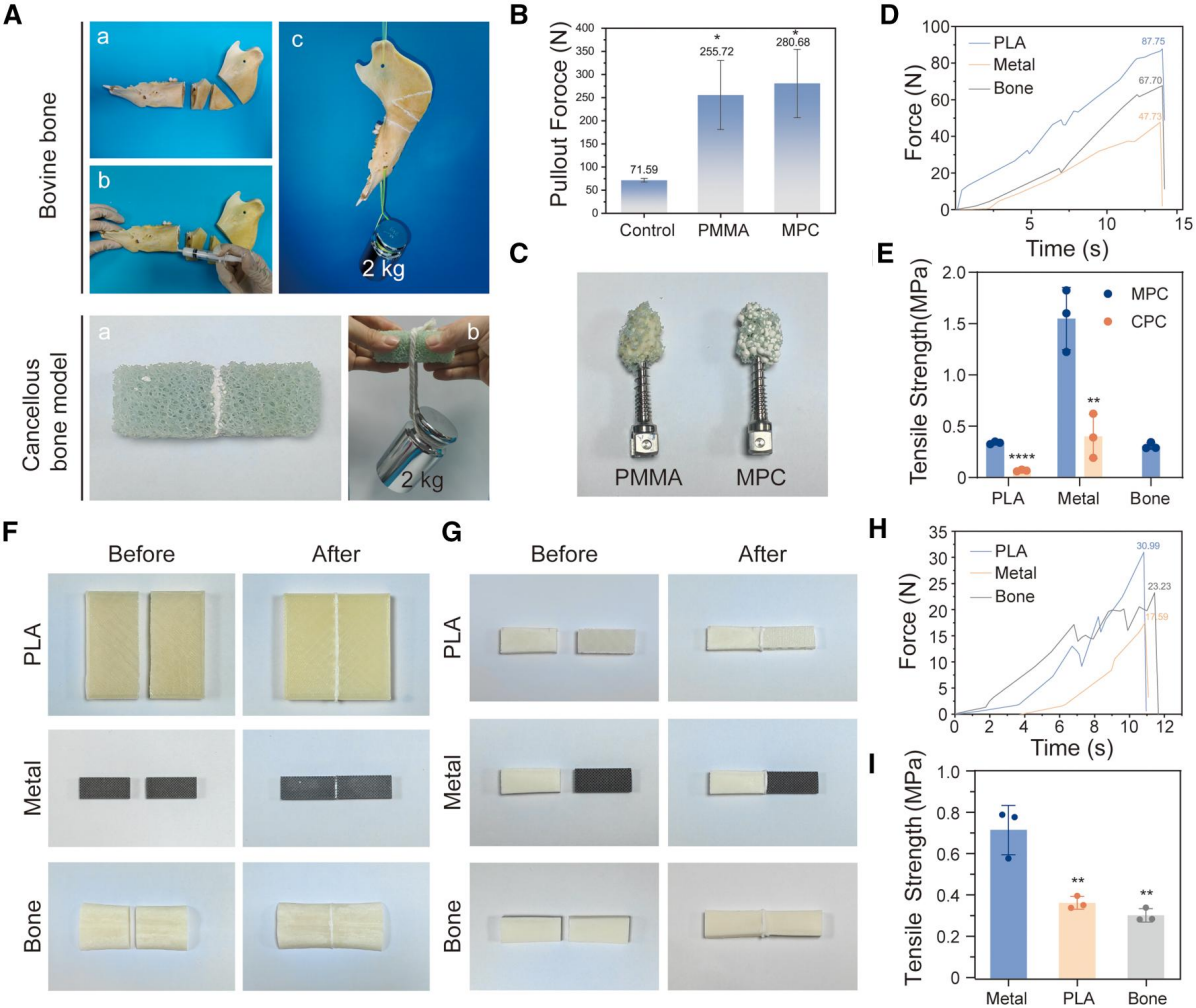
*In vitro* adhesive properties and screw fixation capability of MPC. (**A**) Physical demonstration of MPC-fixed bovine bone and cancellous bone model under a 2 kg load. (**B**) Maximum pull-out force of screws augmented with MPC versus PMMA bone cement (*n* = 4). (**C**) The physical image of removing the screws after fixation with PMMA and MPC screws in the cancellous bone model. (**D**) Time–force curves during pull-out testing of PLA, metal scaffold and bovine bone bonded with MPC bone cement. (**E**) Tensile strength images of MPC-bonded bovine bone, PLA and metal scaffold (*n *= 3). (**F**) Physical images of PLA, metal scaffold and bovine bone before and after bonding with MPC bone cement. (**G**) Separate physical images of the before and after views of the bovine bone–PLA, bovine bone–metal scaffold and bovine bone–bovine bone combinations were obtained using MPC bone cement for adhesion. (**H**) The time–force curves of the bonding of bovine bone–PLA, bovine bone–metal scaffold and bovine bone–bovine bone were obtained using MPC bone cement respectively. (**I**) Tensile strength images of the bonding of bovine bone–PLA, bovine bone–metal scaffold and bovine bone–bovine bone were obtained using MPC bone cement, respectively (*n *= 3). Data presented as mean ± SD; *P < 0.05, **P < 0.01, ****P < 0.0001.

*In vitro* adhesion testing further revealed the exceptional bone bonding properties of MPC. The fractured models of bovine bone and trabecular bone, after being re-bonded with MPC, sustained an axial load of 2 kg (Figure 4A). Both PMMA and MPC significantly enhanced the maximum pull-out force of screws compared to unreinforced bone. During pull-out tests, both cements exhibited higher strength than cancellous bone (Figure 4B). Macroscopic images post-pull-out revealed differing cement dispersion patterns between the two materials, although fracture locations within the cancellous bone model were consistent (Figure 4C). The maximum pull-out force exhibited strong correlation with the dispersion state of the cement, contributing to significant data variability. Statistical analysis (SPSS) indicated no significant difference in pull-out force between the PMMA and MPC groups (*P *= 0.870).

Compared to commercially available CPC, MPC demonstrated significantly superior adhesive strength to metal (tantalum scaffold), organic polymer (PLA) and bovine bone substrates, with statistically significant differences. Specifically, MPC exhibited an average tensile strength of 0.339 MPa on PLA, representing a 5.5-fold increase over CPC. Similarly, MPC’s adhesion strength to the tantalum scaffold was 2.86 times higher than that of CPC (Figure 4E). In bovine bone adhesion tests, MPC achieved an average tensile strength of 0.312 MPa. In stark contrast, CPC provided minimal adhesion; post-hydration, even minor handling caused separation at the bonded bovine bone interface. CPC also displayed pronounced structural disintegration, hindering subsequent mechanical testing. Conversely, MPC formed a stable bonding interface, demonstrating significantly enhanced bonding capability (Supplementary Figure S10). The cut bovine bones were bonded with MPC to PLA, tantalum scaffolds and bovine bones of similar size respectively, all showing good adhesion performance. Among them, the bonding effect on the tantalum scaffold was the best, reaching 0.727 MPa, which showed a significant difference from the pull-out forces of PLA–bovine bone and bovine bone–bovine bone (Figure 4I). These results confirm MPC’s excellent bone fixation properties. This material shows promise for providing reliable fixation in patients with osteoporosis or bone defects, preventing prosthesis loosening or displacement, enhancing implant anchorage and assisting plate or screw fixation.

The primary adhesion mechanism of MPC arises from its rapid hydration reaction generating sparingly soluble magnesium phosphate salts (e.g. K-struvite), which induces immediate system supersaturation and triggers abundant nucleation of micro-/nanoscale acicular or plate-like crystals. These interpenetrating crystals consolidate into a dense, high-strength 3D crystalline network that establishes robust bonding via two complementary pathways, including mechanical interlocking through physical anchorage within bone micro-pores, metallic implant asperities or polymeric interfacial voids, and chemoadhesion where nascent crystal surfaces develop ionic bonds and hydrogen bonding with bone hydroxyapatite, metallic oxide layers or polymeric polar groups. This synergistic dual-action mechanism enables MPC to rapidly integrate disparate substrates—including bone, metal and polymer—into a unified structure within minutes.

### *In vitro* biological performance

**Figure 5:**
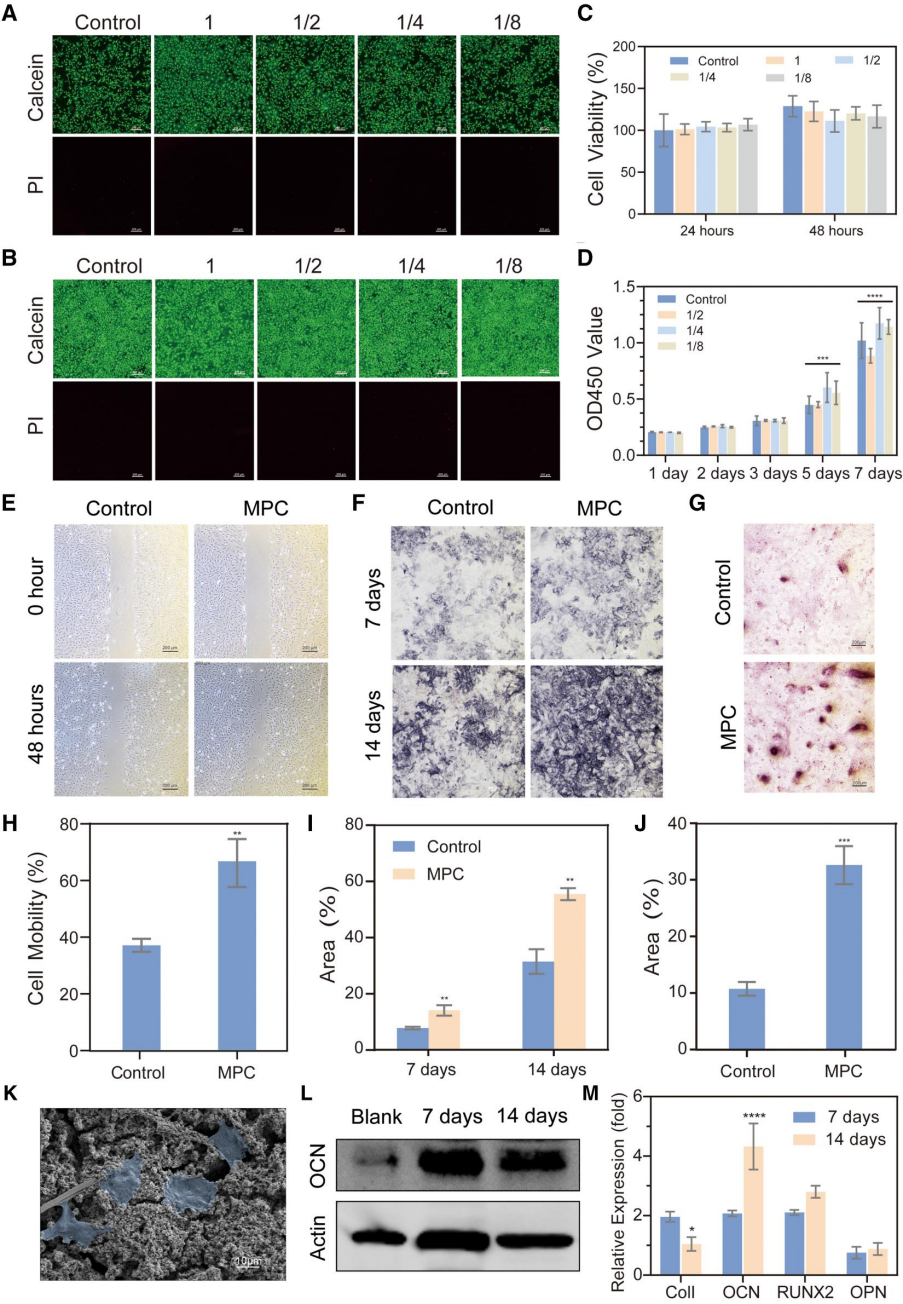
Biocompatibility and osteogenic differentiation of MPC in bone marrow mesenchymal stem cells (BMSCs). (**A**–**B**) Live/dead cell staining of BMSCs cultured with different concentrations of extracts for 24 h (**A**) and 48 h (**B**). The numbers 1, 1/2, 1/4 and 1/8 represent the undiluted extract and 2-fold, 4-fold and 8-fold dilutions, respectively. (**C**) Cytotoxicity of different concentrations of extracts. (**D**) Cell proliferation assay of different concentrations of extracts. (**E** and **H**) Cell migration assay (**E**) and quantitative results (**H**) of blank and MPC groups. (**F** and **I**) Alkaline phosphatase (ALP) staining images (**F**) and quantitative results (**I**) of blank and MPC groups, where dark blue indicates ALP-positive expression. (**G** and **J**) Alizarin red (ARS) staining images (**G**) and quantitative results (**J**) of the blank and MPC groups on the 21st day. (**K**) SEM images of cells co-cultured with MPC materials. (**L**) Western blot results of the MPC groups on days 7 and 14 of co-culture with BMSCs. (**M**) PCR results of the MPC groups on days 7 and 14 of co-culture with BMSCs. Data presented as mean ± SD; **P < 0.01, ***P < 0.001, ****P < 0.0001.

As a bioactive material, MPC demonstrates significant potential for bone repair by releasing Mg^2+^, which exert multifaceted regulatory effects on BMSCs. To evaluate its biocompatibility, MPC extracts at varying concentrations were tested *in vitro*. MTT cytotoxicity assays performed within 24 or 48 h showed that even 100% MPC extract maintained over 80% BMSCs viability (Figure 5C). Live/dead staining using Calcein-AM (green fluorescence for live cells) and propidium iodide (PI, red fluorescence for dead cells) revealed minimal red fluorescence after 24 or 48 h of co-culture, indicating negligible cytotoxicity. Furthermore, increased green fluorescence intensity after 48 h confirmed that MPC does not inhibit cell proliferation, consistent with the MTT results (Figure 5A and B). Actin and DAPI staining further confirmed that BMSCs maintained excellent morphology before and after co-culture with MPC extract, exhibiting typical adherent growth without cell rounding or shrinkage, and maintaining compact cell density with no observable growth inhibition (Supplementary Figure S12). These findings collectively underscore the minimal cytotoxicity and excellent short-term biocompatibility of MPC toward BMSCs. Cell proliferation assays revealed that optical density (OD) values of cells co-cultured with extracts at different concentrations were comparable to those of the blank control group at each tested timepoint, further demonstrating the absence of cytotoxic effects from the bone cement extracts on stem cells (Figure 5D). As critical bioactive ions, calcium (Ca^2+^) and magnesium (Mg^2+^) play pivotal roles in cell migration. Migration assays demonstrated that MPC extract significantly enhanced BMSCs migration rates (Figure 5E and H).

*In vitro* osteogenic induction experiments revealed that exposure to 10 mg/mL MPC extract for 7 and 14 days induced dark blue coloration in ALP staining within some cells, indicating early osteogenic differentiation of BMSCs (Figure 5F and I). During later stages of osteogenesis, calcium ions contribute to the formation of mineralized deposits (‘calcium nodules’). Alizarin red staining at 14 days demonstrated that BMSCs co-cultured with MPC extract produced significantly more calcium nodules than controls, confirming MPC’s promotion of BMSCs mineralization—a critical process for osteogenic differentiation and *in vivo* bone defect remineralization (Figure 5G and J). SEM images of BMSCs co-cultured with MPC revealed robust cell adhesion and growth. On flat scaffold regions, BMSCs exhibited a spread-out, multi-tentacled morphology, while in irregular regions, cells extended pseudopodia to anchor onto the scaffold, suggesting active migration (Figure 5K).

Western blot analysis showed elevated protein levels of osteocalcin (OCN), a key osteogenic differentiation marker, after 7 and 14 days of exposure to MPC extracts at varying concentrations. This indicates that MPC enhances OCN expression to drive differentiation (Figure 5L). Quantitative PCR (qPCR) analysis of osteogenic genes (CoI I, OPN, RUNX2 and OCN) revealed significant upregulation of CoI I, OPN, RUNX2 and OCN after 7 and 14 days of MPC treatment (Figure 5M). These results collectively demonstrate that MPC, primarily through Mg^2+^ release, promotes BMSCs proliferation, migration and osteogenic differentiation, while exhibiting excellent biocompatibility. Its dual functionality as a bioactive scaffold and mineralization inducer positions MPC as a promising candidate for bone regeneration therapies.

### *In vivo* bone regeneration and screw fixation enhancement

**Figure 6:**
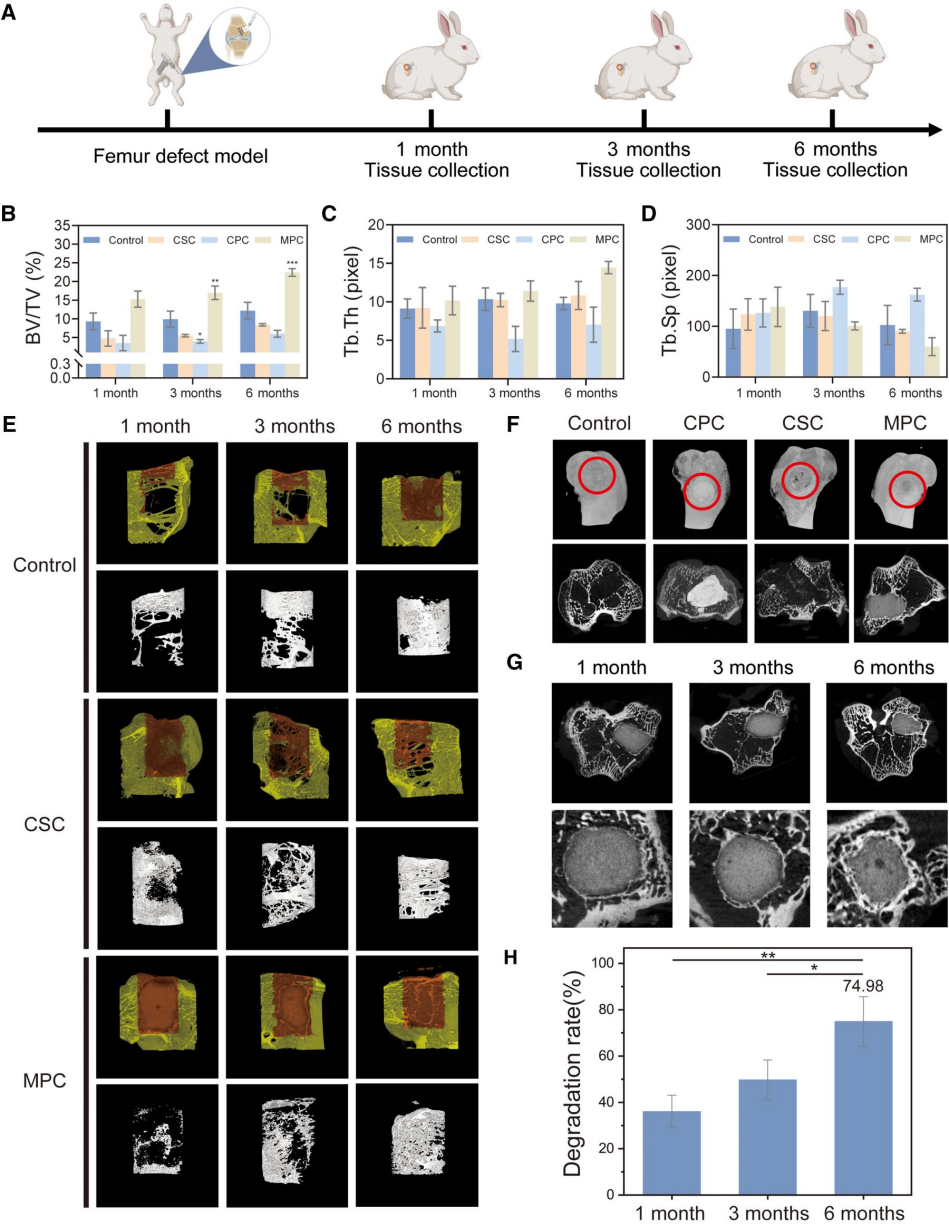
Osteogenic performance of MPC *in vivo* for treating femoral defects. (**A**) Schematic diagram of the animal experimental timeline. (**B–D**) Quantitative analysis of bone volume/tissue volume (BV/TV), trabecular thickness (Tb.Th) and trabecular separation (Tb.Sp) at the defect site at 1, 3 and 6 months post-surgery. (**E**) Three-dimensional (3D) reconstruction images at 1, 3 and 6 months post-surgery showing new bone formation and distribution in different groups. (**F**) 3D reconstruction models and micro-computed tomography (Micro-CT) images of different groups at 3 months post-surgery. (**G**) Micro-CT images of the MPC group at 1, 3 and 6 months post-surgery. (**H**) The *in vivo* degradation rate of MPC. Data presented as mean ± SD; *P < 0.05, **P < 0.01, ***P < 0.001.

The osteogenic capacity of MPC was evaluated using a femoral defect model in New Zealand white rabbits. Specimens were harvested at 1, 3 and 6 months post-implantation for osteogenic analysis (Figure 6A). Micro-CT semiquantitative assessment of new bone formation revealed that at 1 month, the bone volume/total volume (BV/TV) ratio in the MPC group showed no significant difference compared to other groups, likely attributable to undegraded cement occupying the space intended for new bone ingrowth. However, as MPC gradually degraded, new bone formation significantly increased, accompanied by increased trabecular thickness (Tb.Th) and reduced trabecular separation (Tb.Sp). By 6 months, the BV/TV ratio followed the trend MPC > CSC > CPC, with MPC demonstrating statistically significant superiority over the other three groups (Figure 6B–D). The limited new bone volume in the CPC group resulted from its slow degradation rate, while the CSC group resembled the blank control due to rapid CSC degradation, which compromised structural support, hindered hematoma bridging and disrupted stem cell recruitment and osteoblast differentiation. Postoperative blood analyses in the MPC group revealed no signs of anemia, infection or abnormalities in liver/kidney function, electrolytes or metal ion levels, all parameters remaining within normal reference ranges, indicating excellent *in vivo* biocompatibility and safety of MPC (Supplementary Figures S14–S16).

Three-dimensional reconstructions and cross-sectional micro-CT images at 3 months post-implantation (Figure 6E and F) showed persistent defect boundaries with poor new bone regeneration and visible defect morphology in the blank and CSC groups. The CPC group exhibited negligible degradation, while MPC displayed irregular degradation patterns, with new bone forming around the resorbing cement material. Over the observation period, progressive MPC degradation was accompanied by substantial new bone formation, with no interfacial gaps or tissue voids, confirming synchronized material resorption and osteogenesis (Figure 6G). Quantification of residual cement volume revealed a degradation rate approaching 75% at 6 months (Figure 6H).

**Figure 7:**
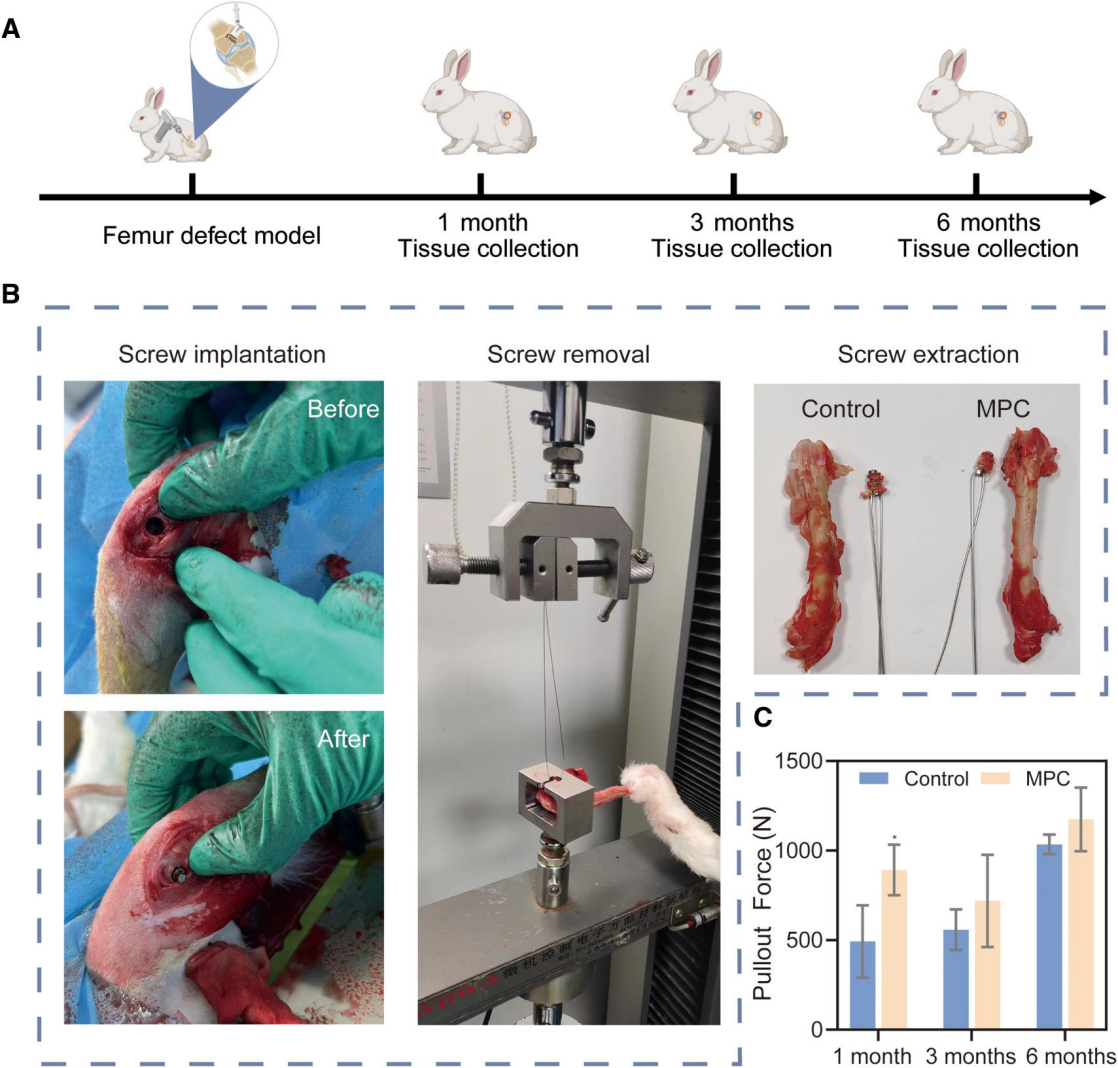
Performance of MPC in vivo for treating screw reinforcement. (**A**) Schematic diagram of the animal experimental timeline. (**B**) Photographs from screw implantation and removal experiments, as well as the actual femur of the rabbit after the screw was removed. (**C**) Screw pullout force in the *in vivo* screw fixation experiment for the blank and MPC groups at 1, 3, and 6 months post-surgery. Data presented as mean ± SD; *P < 0.05.

To evaluate *in vivo* fixation efficacy, a femoral screw fixation model was employed (Figure 7A and B). Results demonstrated that screws augmented with MPC exhibited significantly higher pull-out forces at 1, 3 and 6 months compared to non-augmented controls (blank group), with the difference being more pronounced at earlier time points. The reduced disparity at later stages may reflect natural bone-mediated fixation; nevertheless, the MPC group consistently outperformed the blank control (Figure 7C). When MPC is implanted into the body, it will disperse within the porous structure of cancellous bone. This process forms a ‘cement–bone complex’ centered around the screw, with bone cement and trabecular bone interwoven together. The changes in the pull-out force between the specimens are related to the dispersion of the cement during the injection process, which is also the reason for the significant difference within the experimental group in the *in vivo* pull-out test. The screw fixed with MPC has a higher pull-out resistance after fixation, which is beneficial for initial stability and greatly improves the postoperative rehabilitation effect.

**Figure 8:**
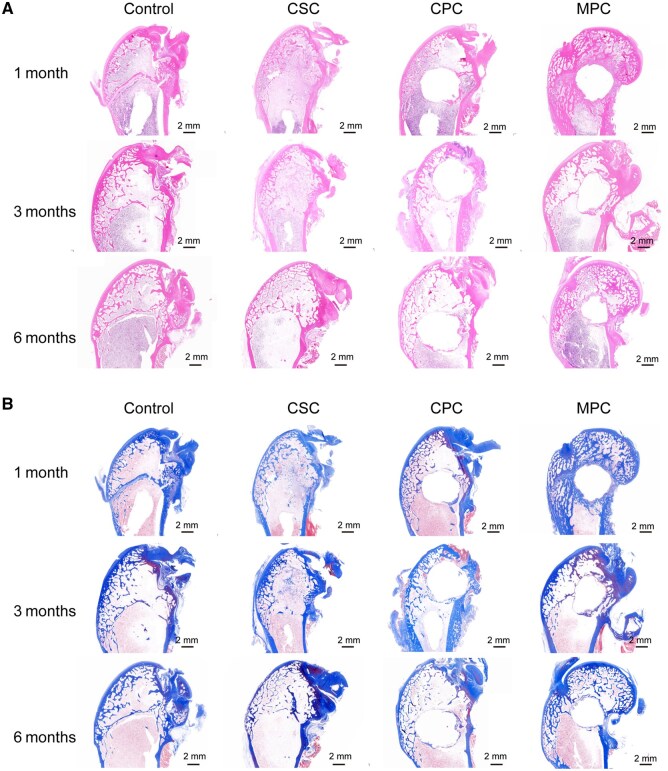
Histological evaluation of bone regeneration and material degradation at femoral defect sites. Representative histological images with (**A**) H&E staining and (**B**) Masson’s trichrome staining of femoral defect regions at 1, 3 and 6 months post-implantation for different groups (blank control, CSC, CPC, MPC).

Subsequent histological staining of femoral sections provided further evidence of MPC’s performance. In the MPC group, H&E staining revealed newly formed bone fuzing into continuous structures that tightly integrated with residual cement fragments, with no voids or inflammatory responses observed. The progressive reduction in cement cross-sectional area confirmed MPC’s favorable *in vivo* degradability. In contrast, CSC degraded completely within 1 month, leaving defects encapsulated by fibrous tissue and forming non-osseous ‘pseudo-joints’. CPC showed minimal degradation, failing to synchronize with bone remodeling. Masson’s trichrome staining further demonstrated significantly superior collagen deposition in the MPC group compared to the other groups (Figure 8A and B), underscoring its exceptional *in vivo* degradability coupled with potent osteogenic efficacy.

## Conclusion

This study establishes MPC as a transformative biomaterial platform that bridges the critical gap between transient mechanical stabilization and sustained biological regeneration in orthopedics. By leveraging the synergistic interplay between MgO and KH_2_PO_4_, we engineered an injectable MPC exhibiting rapid setting kinetics, robust initial compressive strength and precisely tunable degradation synchronized with endogenous bone formation. Crucially, MPC transcends traditional CPC limitations by integrating active biofunctionality. Its controlled degradation releases osteogenic Mg^2+^ ions that orchestrate BMSCs recruitment, proliferation and RUNX2-mediated differentiation, thereby accelerating mineralization. This degradation-guided osteogenesis paradigm ensures complete material resorption, effectively preventing void formation. Progressive micro-CT and histological analyses confirm this process, revealing intimate material–bone integration and near-complete defect regeneration within 6 months. Furthermore, MPC serves as a versatile bioactive fixation enhancer. It exhibits superior adhesion to diverse substrates and significantly augments *in vivo* screw pull-out forces (*P* < 0.05 at early timepoints). This capability directly addresses the critical challenge of implant loosening in osteoporotic or comminuted fractures. The material’s intrinsic safety profiles further underscore its clinical translatability. Unlike bioinert PMMA or slow-degrading CPC, MPC dynamically participates in healing. Its initial mechanical superiority provides immediate stability while subsequent Mg^2+^-driven bioactivity catalyzes microenvironment remodeling. While limitations such as the biological impact of early degradation, unverified fatigue performance and the need for large-animal studies remain, the results of this study robustly demonstrate the potential of a highly bioactive and degradable MPC platform for dual-functional applications in bone defect repair and implant enhancement.

## Supplementary Material

rbaf133_Supplementary_Data
