# Supplementary material for: An all-in-one injectable biocement: self-setting magnesium phosphate for bone repair, fracture adhesion and osteoporotic fixation
Source: Regen Biomater. 2025 Dec 27;13:rbaf133. doi: 10.1093/rb/rbaf133 (PMC12981029; doi:10.1093/rb/rbaf133)
Supplement: rbaf133_Supplementary_Data [file rbaf133_supplementary_data.docx]

**An All-in-One Injectable Biocement: Self-Setting Magnesium Phosphate for Bone Repair, Fracture Adhesion, and Osteoporotic Fixation**

Xinyu Qu^1,a^, Mengting Yin^1,a^, Zhongyi Sun^1,b^, Zhang Liu^a^, Jing Ru^a^, Haibo Liu^a^, Rui Xu^a^, Olga Musskaya^c^, Ilya Glazov^c^, Bingqiang Lu^a^, Xinyu Zhao^a,*^, Bingdi Chen^a,*^, Anatoly Kulak^c,*^, and Feng Chen^a, d*^

^a^Center for Orthopaedic Science and Translational Medicine, Department of Orthopedics, Shanghai Tenth People’s Hospital, School of Medicine, Tongji University, Shanghai 200072, P. R. China

^b^Department of Orthopedics, Changzheng Hospital, Second Military Medical University (Naval Medical University), Shanghai 200003, P. R. China

^c^Institute of General and Inorganic Chemistry, National Academy of Sciences of Belarus, Minsk 220072, Belarus

^d^Suzhou First People's Hospital, School of Medicine, Anhui University of Science and Technology, Anhui 232001, P.R. China

These authors contributed equally to this work.

*Corresponding author:

Xinyu Zhao, xyzhao@tongji.edu.cn; Bingdi Chen, inanochen@tongji.edu.cn; Anatoly Kulak, kulak@igic.bas-net.by; Feng Chen, fchen@tongji.edu.cn


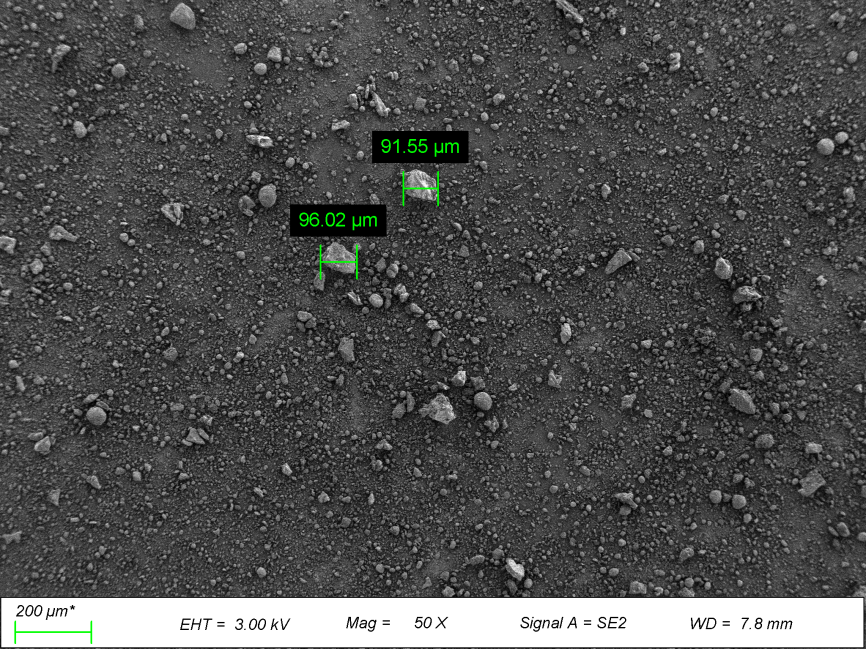


Supplementary Fig 1. SEM images of mixed solid-phase powder.





Supplementary Fig 2. SEM image of the tightly adhered outer surface of MPC


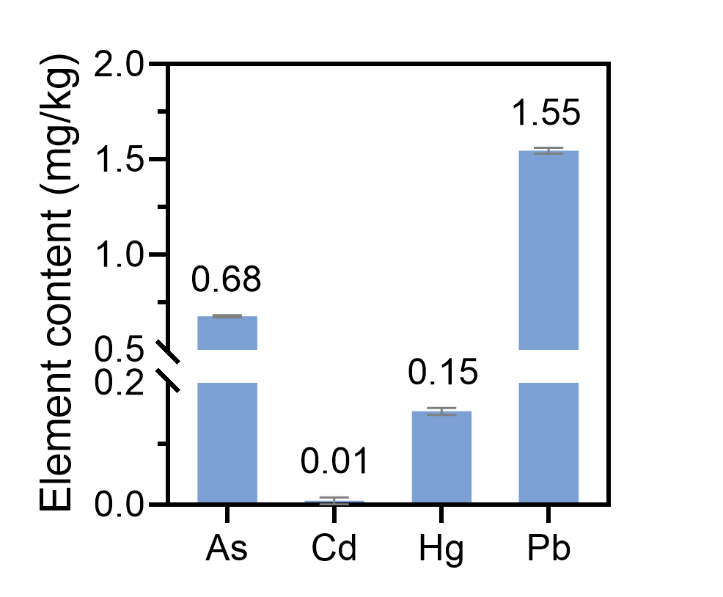


Supplementary Fig 3. Heavy metal content confirming biocompatibility thresholds (<5 ppm, n = 3).

Supplementary Table 1. Porosity analysis of MPC by Mercury Intrusion Porosimetry.

| Intrusion Data Summary | |
| --- | --- |
| Total Intrusion Volume | 0.0212 mL/g |
| Median Pore Diameter (Volume) | 11050.9 nm |
| Median Pore Diameter (Area) | 12.9 nm |
| Average Pore Diameter (4V/A) | 157.2 nm |
| Bulk Density at 0.52 psia | 1.9371 g/mL |
| Apparent (skeletal) Density | 2.0200 g/mL |
| Porosity | 4.1015% |


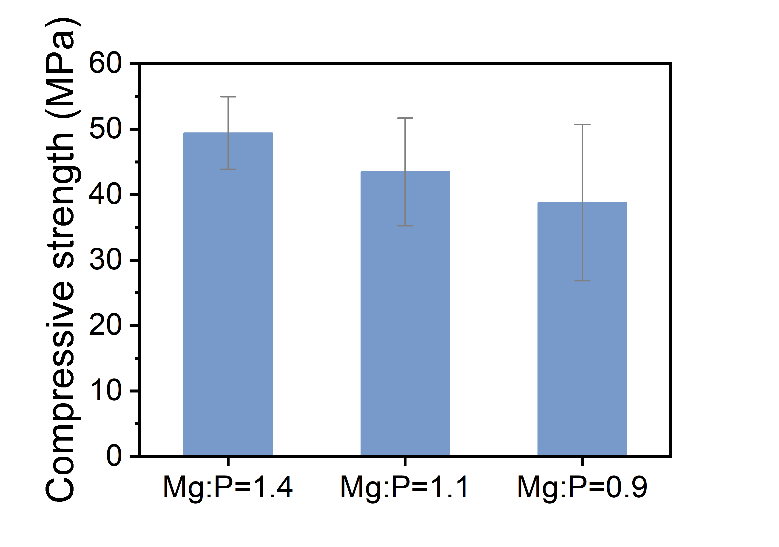


Supplementary Fig 4. Effect of Mg/P ratio on the compressive strength of MPC.


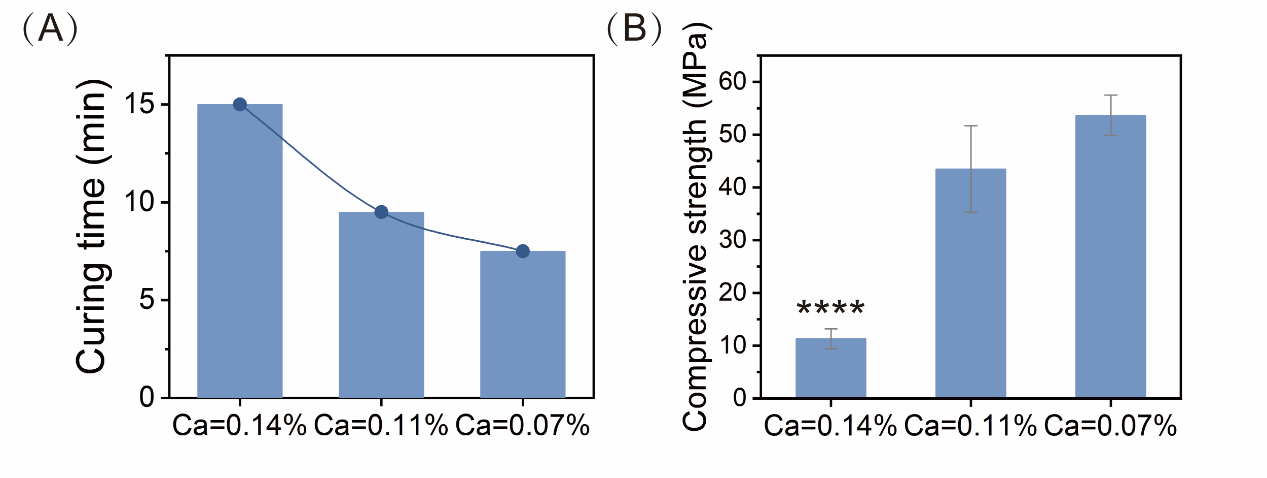


Supplementary Fig 5. Effect of Ca(H_2_PO_4_)_2_ content on the curing time(A) and compressive strength(B) of MPC.


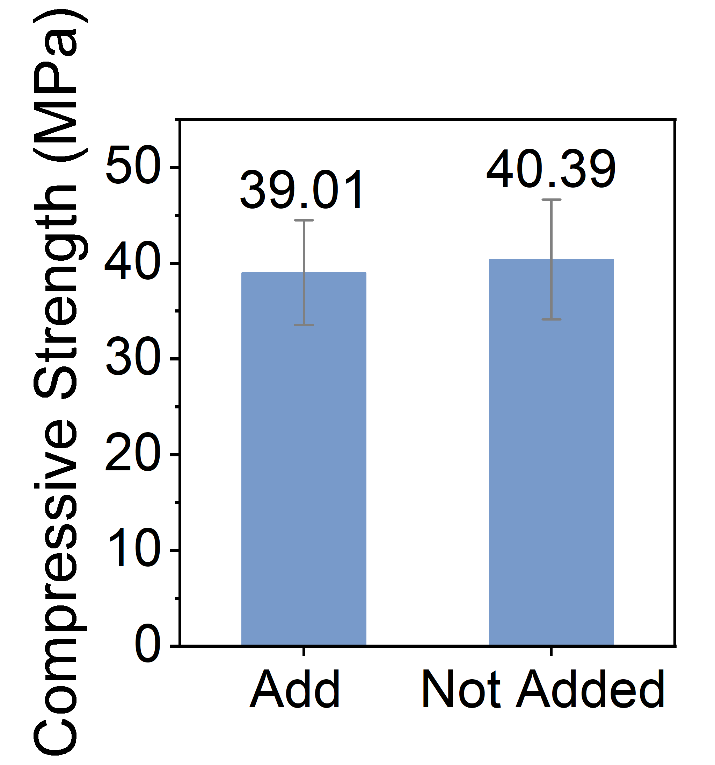


Supplementary Fig 6. Effect of sucrose addition on compressive strength (n= 6).


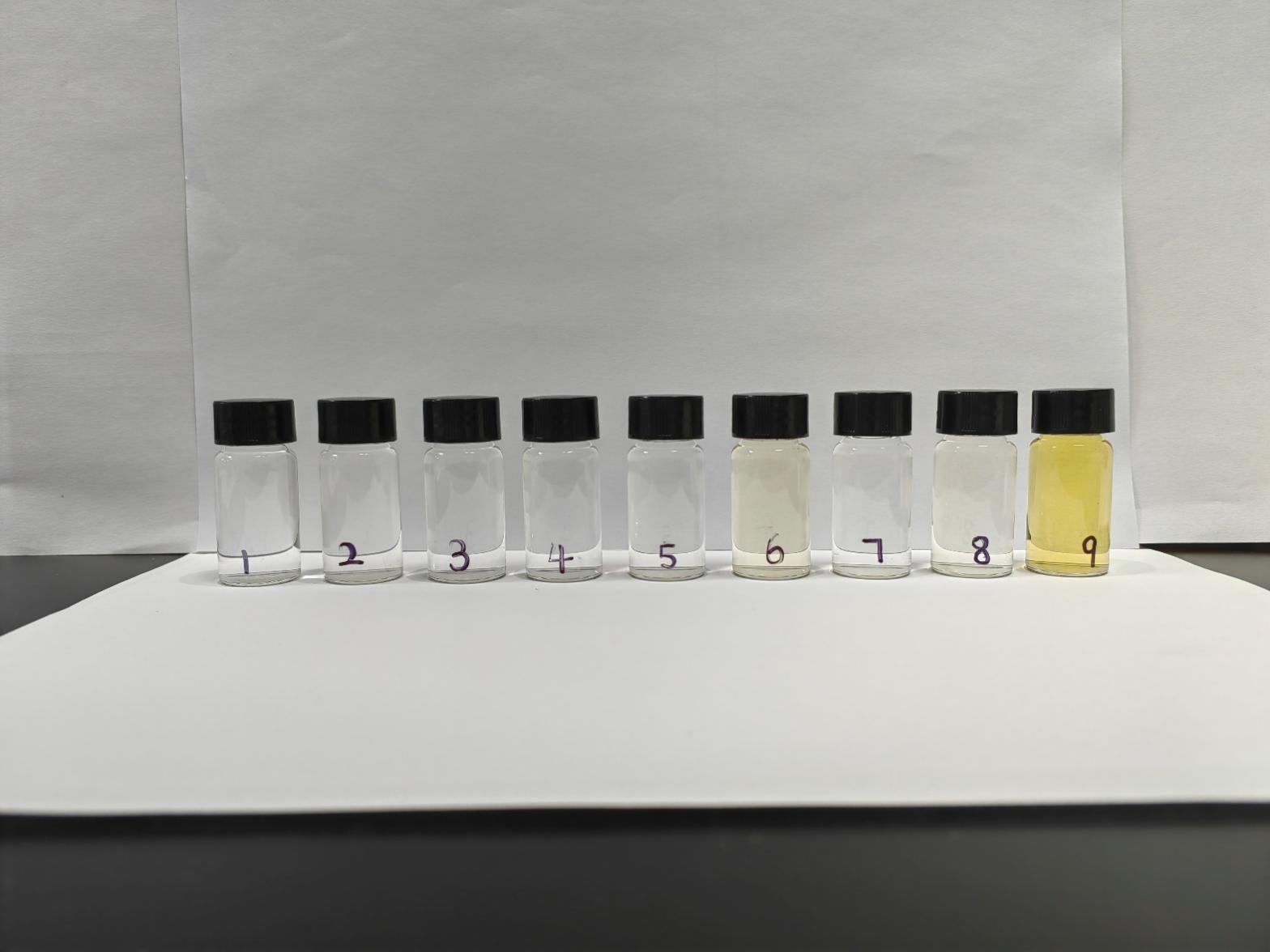


Supplementary Fig 7. Physical images of the liquid-phase after different processing methods. From left to right: citric acid solution, sucrose solution, citric acid and sucrose mixed solution, irradiated citric acid solution, irradiated sucrose solution, irradiated citric acid and sucrose mixed solution, citric acid solution sterilized by autoclaving, sucrose solution sterilized by autoclaving, and citric acid and sucrose mixed solution sterilized by autoclaving.


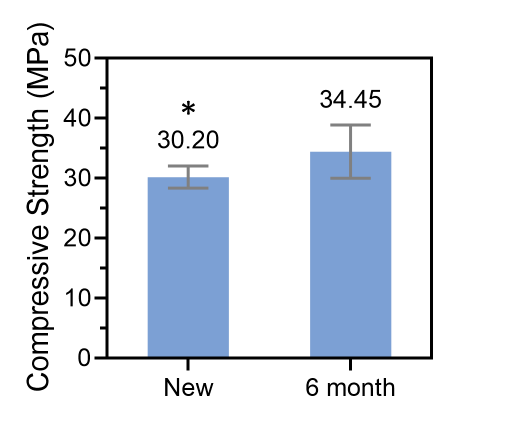


Supplementary Fig 8. Effect of 6-month-stored citric acid solution on the compressive strength of MPC.


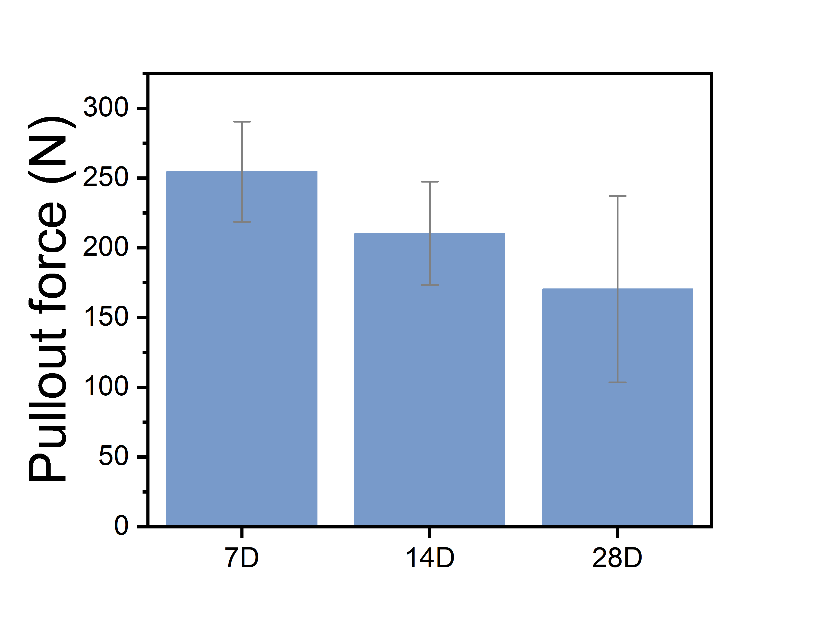


Supplementary Fig 9. Pullout force of screws fixed with MPC after immersion at 37°C for different time.


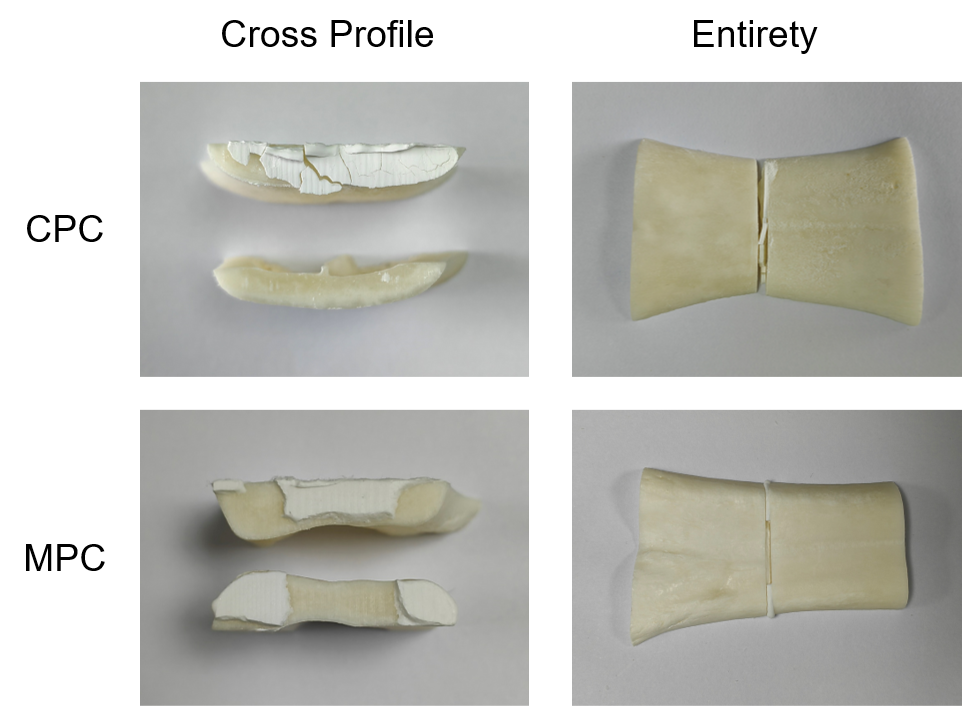


Supplementary Fig 10. Cross-profile and entirety views of bovine bone bonded with MPC versus CPC.


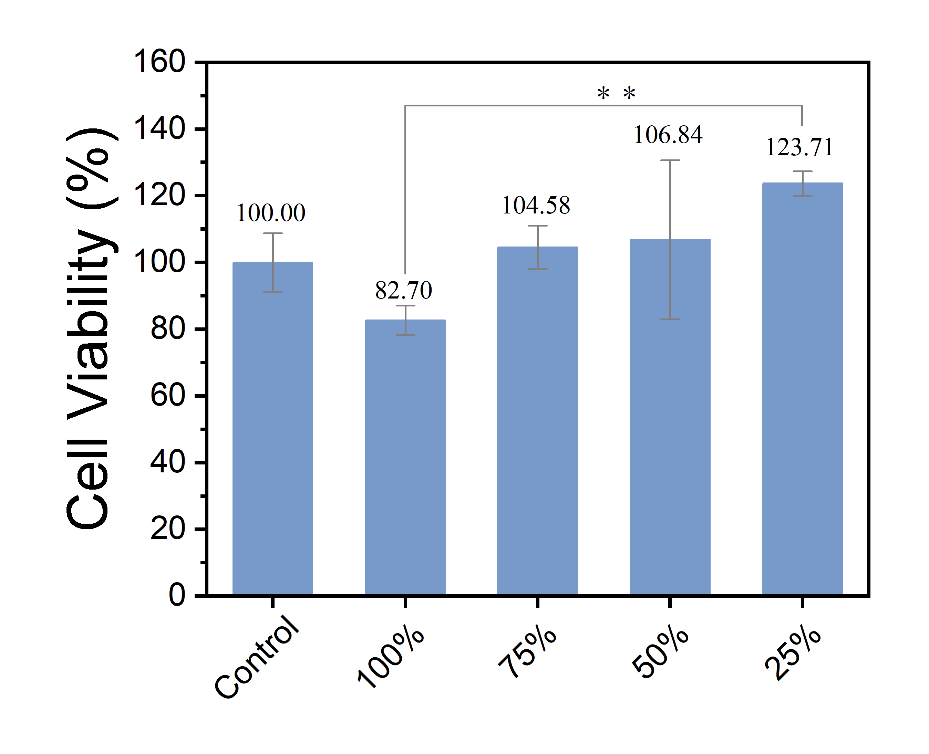


Supplementary Fig 11. Cytotoxicity evaluation of L-929 cells co-cultured with MPC extracts at different concentrations for 24 hours.


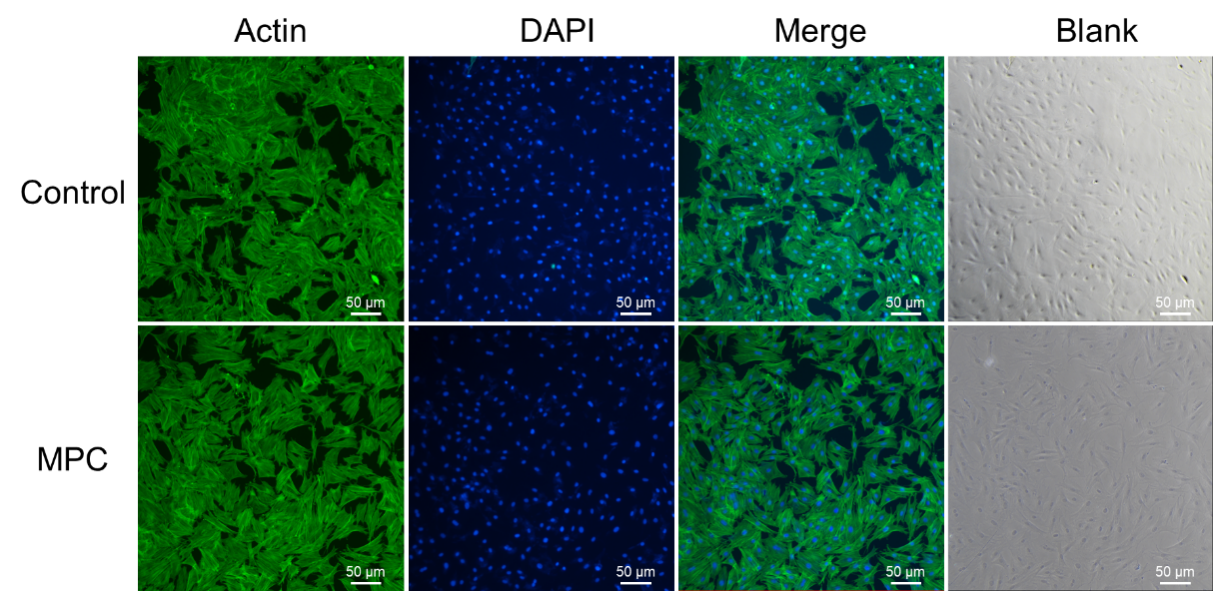


Supplementary Fig 12. Cytoskeletal staining images of BMSCs co-cultured with MPC extracts.


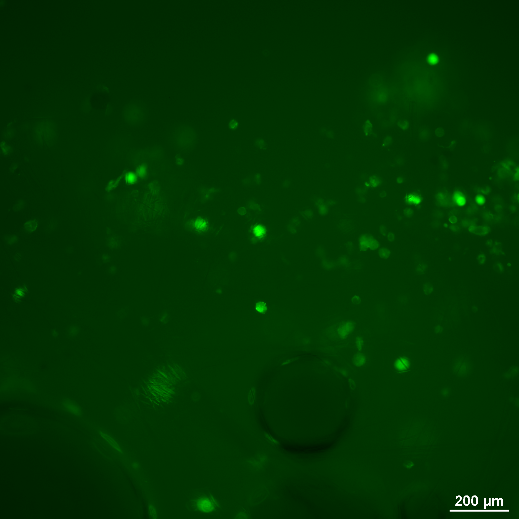


Supplementary Fig 13. Calcein-AM fluorescence staining of BMSCs co-cultured with MPC for 24 hours.

Supplementary Table 2. q-PCR primers.

| Primers | Oligo sequences 5’-3’ |
| --- | --- |
| Osteocalcin-forward  Osteocalcin-reverse | GCTGCCCTAAAGCCAAACTCT  AGAGGACAGGGAGGATCAAGTTC |
| Osteopontin-forward  Osteopontin-reverse | AGCAAGAAACTCTTCCAAGCAA  GTGAGATTCGTCAGATTCATCCG |
| Runx2-forward  Runx2-reverse | CCGCACGACAACCGCACCAT  CGCTCCGGCCCACAAATCTC |
| Type I collagen-forward  Type I collagen-reverse | GCAACAGTCGCTTCACCTACA  CAATGTCCAAGGGAGCCACAT |
| GAPDH-forward  GAPDH-reverse | TCTCTGCTCCTCCTGTTCGA  GCGCCCAATACGACCAAATC |


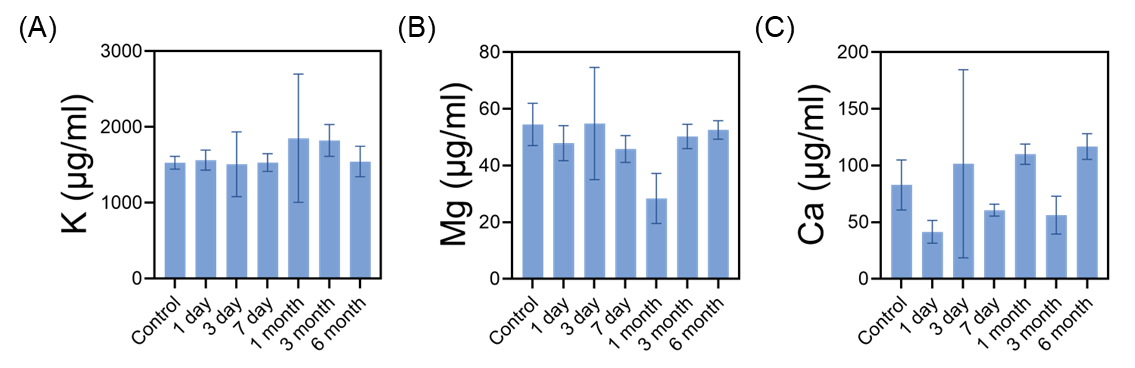


Supplementary Fig 14. Concentrations of K (A), Mg (B), and Ca (C) in rabbit marginal ear vein blood at different postoperative timepoints.
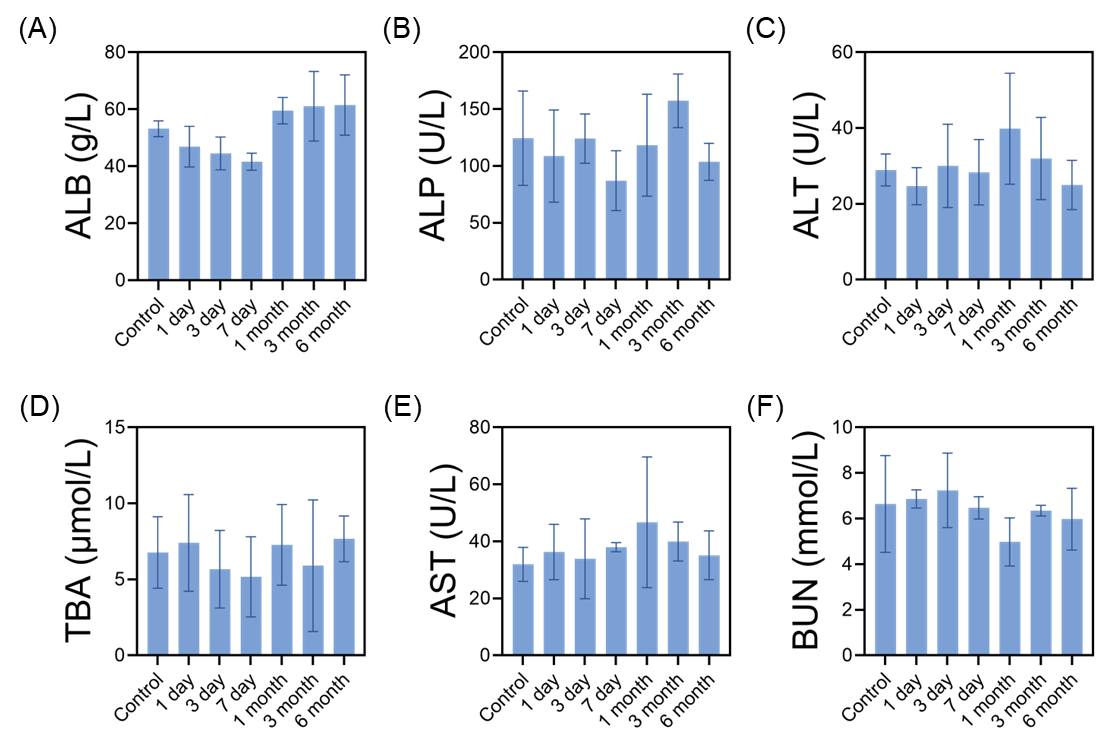


Supplementary Fig 15. Analysis of ALB (A), ALP (B), ALT (C), TBA (D), AST (E) and BUN (F) concentrations in rabbit marginal ear vein blood at different postoperative timepoints.


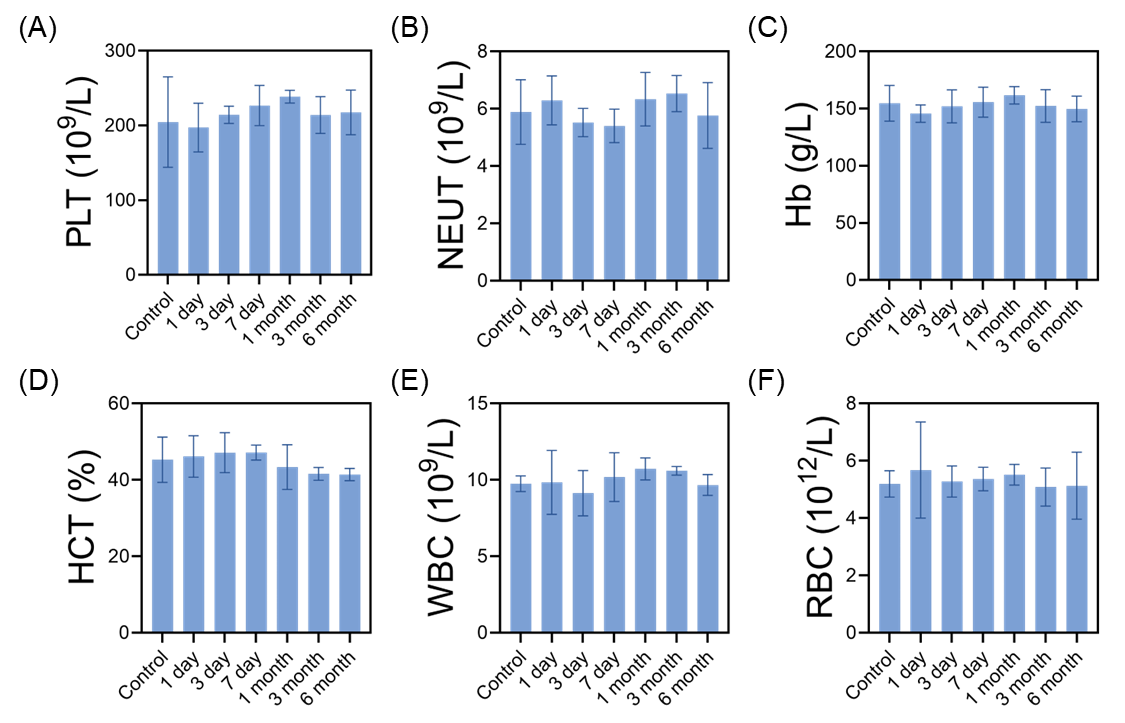


Supplementary Fig 16. Analysis of PLT (A), NEU (B), Hb (C), HCT (D), WBC (E) and RBC (F) levels in rabbit marginal ear vein blood at different postoperative timepoints.
